# Niclosamide Revitalizes Sorafenib through Insulin-like Growth Factor 1 Receptor (IGF-1R)/Stemness and Metabolic Changes in Hepatocellular Carcinoma

**DOI:** 10.3390/cancers15030931

**Published:** 2023-02-01

**Authors:** Syue-Wei Peng, Mai-Huong T. Ngo, Yung-Che Kuo, Ming-Hao Teng, Chin-Lin Guo, Hung-Cheng Lai, Te-Sheng Chang, Yen-Hua Huang

**Affiliations:** 1 Graduate Institute of Medical Sciences, College of Medicine, Taipei Medical University, Taipei 11031, Taiwan; 2 Department of Biochemistry and Molecular Cell Biology, School of Medicine, College of Medicine, Taipei Medical University, Taipei 11031, Taiwan; 3 TMU Research Center for Cell Therapy and Regeneration Medicine, Taipei Medical University, Taipei 11031, Taiwan; 4 Institute of Physics, Academia Sinica, Taipei 11529, Taiwan; 5 Department of Obstetrics and Gynecology, Shuang Ho Hospital, Taipei Medical University, New Taipei City 23561, Taiwan; 6 Department of Obstetrics and Gynecology, School of Medicine, College of Medicine, Taipei Medical University, Taipei 11031, Taiwan; 7 School of Traditional Chinese Medicine, College of Medicine, Chang Gung University, Taoyuan 33382, Taiwan; 8 Division of Gastroenterology and Hepatology, Department of Internal Medicine, Chang Gung Memorial Hospital, Chiayi 61363, Taiwan; 9 Center for Reproductive Medicine, Taipei Medical University Hospital, Taipei Medical University, Taipei 11031, Taiwan; 10 International PhD Program for Cell Therapy and Regeneration Medicine, College of Medicine, Taipei Medical University, Taipei 11031, Taiwan; 11 PhD Program for Translational Medicine, College of Medical Science and Technology, Taipei Medical University, Taipei 11031, Taiwan

**Keywords:** HCC sorafenib resensitization, IGF-1R, metabolic change, niclosamide, organoid

## Abstract

**Simple Summary:**

Sorafenib resistance is the major challenge for the unsatisfactory response to targeted therapies in advanced hepatocellular carcinoma (HCC). Combination therapy may overcome the obstacle and is an increasingly acceptable strategy. In this study, we demonstrate that niclosamide can increase sorafenib sensitivity in sorafenib-resistant HCC cells/organoids through the regulation of insulin-like growth factor 1 receptor (IGF-1R)/p-IGF-1R/stemness and metabolic changes. A combination of sorafenib and niclosamide can yield a synergistic combination index (CI) for sorafenib-resistant HCC cells that decrease the expressions of IGF-1R/p-IGF-1R/OCT4, attenuate stemness-related properties, downregulate the glycolysis-associated gene expressions, and reduce the mitochondrial membrane potential in vitro, and decrease tumor size and tumor volume resulting from apoptosis in vivo. These findings may highlight a practical clinical strategy for repurposing niclosamide for enhancing sorafenib sensitivity in HCC.

**Abstract:**

Sorafenib is the first approved systemic targeting agent for advanced HCC; however, when used alone, drug resistance can result in considerably reduced efficacy. Here, we demonstrate that niclosamide, an antihelminthic agent approved by the US Food and Drug Administration, can be repurposed to increase sorafenib sensitivity in sorafenib-resistant HCC cells. We generated sorafenib-resistant HCC cell lines (HepG2215_R and Hep3B_R) with elevated IGF-1R levels and strong properties in terms of stemness and epithelial–mesenchymal transition. Niclosamide was found to increase sorafenib sensitivity effectively in both cell lines and their organoids. The underlying mechanism involves the modulation of cancer stemness, IGF-1R/p-IGF1R/OCT4, and metabolic changes. The combination of sorafenib and niclosamide, but not linsitinib, effectively suppressed the IGF-1R/OCT4 expressions, yielded a synergistic combination index (CI), and attenuated stemness-related properties such as secondary tumor sphere formation and cell migration in sorafenib-resistant HCC cells. Notably, niclosamide significantly suppressed the sorafenib-induced IGF-1R phosphorylation prompted by IGF-1 treatment. Niclosamide effectively downregulated the sorafenib-induced gene expression associated with glycolysis (*GLUT1*, *HK2*, *LDHA*, and *PEPCK*), stemness (*OCT4*), and drug resistance (*ABCG2*) and enhanced the ability of sorafenib to reduce the mitochondrial membrane potential in vitro. The synergistic effect of a combination of niclosamide and sorafenib in vivo was further demonstrated by the decreased tumor size and tumor volume resulting from apoptosis regulation. Our results suggest that niclosamide can enhance sorafenib sensitivity in sorafenib-resistant HCC cells through IGF-1R/stemness regulation and metabolic changes. Our findings highlight a practical clinical strategy for enhancing sorafenib sensitivity in HCC.

## 1. Introduction

Hepatocellular carcinoma (HCC), which is highly endemic in Asia and Africa, is the sixth most common cancer and the third leading cause of cancer mortality worldwide [1]. Despite well-established knowledge of etiologies and the availability of effective prevention strategies, the efficacy of clinical treatment for HCC remains unsatisfactory [2,3]. Surgical resection or local ablation is the gold standard treatment for early-stage HCC [4]. However, tumor recurrence is common, with 3-year and 5-year disease-free survival rates of only 50% and 36%, respectively [5]. For advanced-stage HCC for which curative local therapies are infeasible, systemic pharmacological therapy is the mainstay of treatment [4]. Although HCC treatment has advanced considerably with various new agents becoming available, the overall patient survival rate is unsatisfactory, indicating a large unmet need for more efficient and affordable therapeutic strategies [6].

Sorafenib (Nexavar) is the first approved systemic targeted therapy agent for unresectable HCC, and despite novel anti-HCC drugs being developed over the past 2 decades, sorafenib has remained a dominant pharmaceutical regimen with a proven survival benefit [6,7,8]. In the SHARP and sorafenib Asia-Pacific trials, patients who received sorafenib treatment obtained significant but clinically limited benefits, as indicated by a 2- to 3-month increase in overall survival compared with placebo [7,8]. Furthermore, approximately 70% of patients with HCC who received adjuvant sorafenib treatment after surgical resection or local ablation (or both) developed tumor recurrence within 5 years postsurgery, and most of these recurrent HCCs were sorafenib refractory [9].

New targeted therapeutic agents including lenvatinib [10], regorafenib [11], and cabozantinib [12] have been approved as first-line or second-line therapies for advanced-stage HCC. However, drug resistance is a major obstacle and none of these drugs has been proven to be superior to sorafenib. Sorafenib is a multitarget kinase inhibitor that blocks cell proliferation through the inhibition of Raf-1, B-Raf, and kinases in the Ras/Raf/MEK/ERK signaling pathway. Sorafenib also inhibits angiogenesis by targeting hepatocyte factor receptors, FMS like tyrosine kinase, vascular endothelial growth factor receptor (VEGFR)-2, VEGF-3, platelet-derived growth factor receptor, and other tyrosine kinases. Several mechanisms contribute to acquired refractoriness to sorafenib, including crosstalk between the PI3K/Akt and JAK-STAT pathways, activation of hypoxia-inducible pathways, epithelial–mesenchymal transition, and enrichment of tumor-initiating cell population [13].

For unresectable HCC, emerging clinical trial results have indicated superior clinical outcomes for combination therapies—including atezolizumab (anti-PD-L1 Ab) + bevacizumab (anti-VEGF Ab) (IMbrave 150) [14] and durvalumab (anti-PD-L1 Ab) + tremelimumab (anti-CTLA4 Ab) (HIMALAYA) [15]—relative to monotherapy; however, sorafenib remains a key regimen alone or in combination with other therapeutic modalities for unresectable HCC especially when sorafenib drug resistance could be overcome [16].

Biochemical differences between cancerous and noncancerous cells may help guide targeted therapies against the malignant elements. Instead of employing mitochondrial oxidative phosphorylation, which is efficient and generates more adenosine triphosphate (ATP) than glycolysis in normal cells, cancer cells rely primarily on aerobic glycolysis [17]. Niclosamide was originally developed as a molluscicide to kill snails but was later approved by the US Food and Drug Administration for use in humans to treat tapeworm infection. Growing evidence has indicated that niclosamide is a multifunctional drug that may interfere with multiple signaling pathways and biological processes, suggesting its potential as a novel treatment for diseases other than helminthic disease [18]. Niclosamide has been reported to suppress the tumor growth of various cancers, including drug-resistant HCC [19], prostate cancer [20], and esophageal cancer [21]. The major antihelminthic mechanism of niclosamide involves the inhibition of mitochondrial oxidative phosphorylation in the cells of parasitic worms, thereby restricting ATP production [7]. Moreover, a study suggested that sorafenib resistance is partially derived from enhanced glycolysis and its cancer cell-killing capacity can be synergized by glycolysis blockade [22].

This study contributes to the potential clinical application by examining the repurposing of niclosamide to increase sorafenib sensitivity in HCC. Relative to conventional drug development, drug repositioning is considerably less expensive and has a shorter approval process. Our results indicated that niclosamide can enhance sorafenib sensitivity in sorafenib-resistant HCC cells through the p-IGF-1R/insulin-like growth factor 1 receptor (IGF-1R)/stemness regulation and metabolic changes. The combination of niclosamide and sorafenib may represent a practical clinical strategy for increasing the sorafenib sensitivity of HCC.

## 2. Materials and Methods

### 2.1. Cell Lines

Hep3B cells (HBV^+^/HBsAg^+^ human HCC, HB-8064) were purchased from the American Type Culture Collection (Manassas, VA, USA) and HepG2215 cells (HBV^+^/HBsAg^+^ human hepatoblastoma) were kindly provided by Dr. Jun-Jen Liu (Institute of Medical Biotechnology, Taipei Medical University, Taipei, Taiwan). These cell lines were maintained in Dulbecco’s modified Eagle medium (DMEM, Gibco-BRL, Thermo Fisher Scientific, Waltham, MA, USA) with 10% fetal bovine serum, 3.7 g/L sodium bicarbonate (Sigma-Aldrich, St. Louis, MO, USA), 1% penicillin–streptomycin (PS, Gibco, Grand Island, NY, USA), and 1% glutamate (Gibco) in a humidified incubator in 5% CO_2._ Sorafenib-resistant cells (HepG2215_R and Hep3B_R) were generated by treating naïve cells (HepG2215 and Hep3B) with low to high concentrations of sorafenib (Cell Signaling, Danvers, MA, USA) from low to high concentrations for continuing 3 months. And sorafenib-resistant cells were maintained in 10 µM sorafenib with the medium as mentioned above. The cell line protocol was the same as the published study by Yen-Hua Huang’s lab [23].

### 2.2. RNA Isolation and Real-Time Reverse-Transcription Polymerase Chain Reaction

The total RNA of the cell was extracted from cells using the EasyPure Total RNA Spin Kit (Bioman Scientific, New Taipei, Taiwan) according to the manufacturer’s directions. The cDNA synthesis was using MMLV reverse transcriptase (Invitrogen, Carlsbad, CA, USA). For the real-time polymerase chain reaction (PCR), PCR amplification was performed with Fast SYBR Green Master Mix (Thermo Fisher Scientific). Primer sequences are listed in Supplementary Table S1. Real-time quantitative RT-PCR analysis of at least three independent cultures was performed in all experiments. The StepOnePlus Real-Time PCR system (Applied Biosystems, Vilnius, Lithuania) condition was the same as the published study by Yen-Hua Huang’s lab [23].

### 2.3. Western Blot Analysis

Cell extracts were prepared with lysis buffer (10 mM Tris [Ph7.5], 150 mM NaCl, 1 mM EDTA, 0.5% sodium deoxycholate, 0.5% NP-40, and 0.1% SDS) plus a protease inhibitor cocktail (Roche Diagnostics, NA, USA) and phosphatase inhibitor cocktail (Roche). The protein concentration was determined using the Pierce BCA assay kit (Thermo Fisher Scientific) according to the manufacturer’s instructions. Equal amounts of total protein were loaded into 10–12% sodium dodecyl sulfate–polyacrylamide gel electrophoresis and transferred to nitrocellulose membranes (Immobilon^®^-P PVDF Membrane, Merck Millipore, Darmstadt, Germany). PVDF membranes were blocked with 5% BSA and then incubated with specific primary antibodies. The antibodies were described in Supplementary Table S2. The signal was detected by the ImageQuant LAS 4000 mini system (GE Healthcare, Chicago, IL, USA). The western blot analysis protocol was the same as the published study by Yen-Hua Huang’s lab [23].

### 2.4. HCC Organoid Generation

A total of 3000 Hep3B_R and HepG2215_R cells were seeded in an ultralow attachment surface polystyrene 6-well plate (Costar^®^, Product Number: 3471, USA) with DMEM-F12 (Gibco™, DMEM/F-12, powder, USA) medium. The additional supplements were as follows: 2% B27 supplement, minus vitamin A (Invitrogen), 1% Glutamax (Thermo Scientific), 5 μM A83-01 (Sigma-Aldrich), 1 μL/mL n-acetyl-L-cysteine (Sigma-Aldrich), 10 mM nicotinamide (Sigma-Aldrich), 0.3 μL/mL CHIR 99021 (Sigma-Aldrich), 20 ng/mL recombinant human epithelial growth factor, EGF (Life Technologies), 50 ng/mL human recombinant fibroblast growth factor, FGF10 (Sigma-Aldrich), and 5 μM γ-27632 dihydrochloride (MedChemExpress). The γ-27632 dihydrochloride was removed from the organoid culture medium after 7 days of culture. The media were replenished every 2 to 3 days. The organoids were harvested after culturing for 14 days. For the WST-1 assay, the organoids were cultivated in Matrigel (Sigma-Aldrich) by seeding a drop in a 48-well plate. A total of 10^3^ cells were mixed with 50 μL Matrigel. Organoids were cultivated for 14 days.

### 2.5. Cell Viability Assay

For proliferation assay of the 2D cells, the naïve or sorafenib-resistant cells were seeded in 96- well plates at 5000 cells/well and incubated at 37 °C in 5% CO_2_ for 24 h or 48 h. For the drug sensitivity assay, the cells were seeded for 24 h and treated with various concentrations of sorafenib (#8705 Cell Signaling) and these cells were then incubated at 37 °C in 5% CO_2_ for 48 h. The 3D organoids were cultivated in Matrigel (Sigma-Aldrich) by seeding a drop in a 48-well plate. A total of 10^3^ cells were mixed with 50 μL Matrigel. Organoids were cultivated for 14 days. Thereafter, a WST-1 assay (Roche) was used to detect cell proliferation according to the manufacturer’s instructions. Three experiments were performed for each experimental condition. Cell viability is expressed as the percentage of non-treated cells.

### 2.6. Mitochondria Membrane Potential Assay

Cells were incubated in DMEM medium containing JC-1 (2 μg/mL, #1130-5, Biovision, Milpitas, CA, USA) at 37 °C for 30 min. The distribution of JC-1 monomers (green fluorescence) and J-aggregate (red fluorescence) fluorescence was captured using a fluorescence microscope. Furthermore, cells were trypsinized and resuspended in phosphate-buffered saline (PBS). Cells were transferred to a 1.5-mL plastic cuvette, and their light emission at 590 (aggregates) and 530 (monomers) nm wavelengths were determined using flow cytometry analysis.

### 2.7. Tumor Xenograft Mouse Model

Eight-week-old NOD/SCID mice (MGI:2163032, National Laboratory Animal Center, Taipei, Taiwan) were subcutaneously inoculated with HepG2215-R cells. The mice have subcutaneously injected with 5 × 10^6^ cells. Sorafenib was administered twice a week. After 5 weeks of implantation, the mice were sacrificed. Tumor tissues were collected and embedded in paraffin wax. The mice were not randomized into groups because this was deemed irrelevant to this study. The animal study protocol was approved by the Institutional Animal Care and Use Committee/Panel at Taipei Medical University, Taipei, Taiwan (Approval number: LAC-2016-0386).

### 2.8. TUNEL Assay

The DNA strand breaks in apoptotic cells were evaluated using the in situ cell death detection kit-a based on TUNEL technology (Roche). In brief, the xenograft paraffin-embedded tissue sections were subjected to the common procedures of immunohistochemistry (dewaxation, rehydration, protease treatment, and permeabilization), as described in the preceding section. After the permeabilization, the tissue sections were rinsed in PBS. The TUNEL reaction mixture was applied to the slides, which were then incubated at 37 °C in a humidified atmosphere, avoiding light, for 60 min. Subsequently, the samples were analyzed under a fluorescence microscope.

### 2.9. Drug Combination Index

Cells were seeded into 96-well plates (BD Falcon, Durham, NC, USA) at 5000 cells/well. Cells were treated with a single drug or a combination. The concentrations of sorafenib were 0.5, 2.5, 5, 10, and 15 µM. The concentrations of niclosamide were 1, 3, 5, 10, 15, 20, 30, and 40 µM. Cell viability was measured using the WST-1 assay. The drug combination index (CI) value was analyzed using Compusyn software (Compusyn, Paramus, NJ, USA). Drug CI values of <0.9, 0.9–1.1, and >1.1 represented synergism, additive effects, and antagonism, respectively.

### 2.10. Statistical Analyses

All experiments were repeated at least three times. The results are presented as the mean ± the standard deviation (SD) as appropriate and were analyzed using Student’s *t*-test and one-way ANOVA (GraphPad Software, La Jolla, CA, USA). A *p*-value of <0.05 was considered significant.

## 3. Results

### 3.1. Sorafenib-Resistant HCC Cells Exhibited Higher IGF-1R Expression Levels and Stronger Stemness/EMT-Related Properties

**Figure 1:**
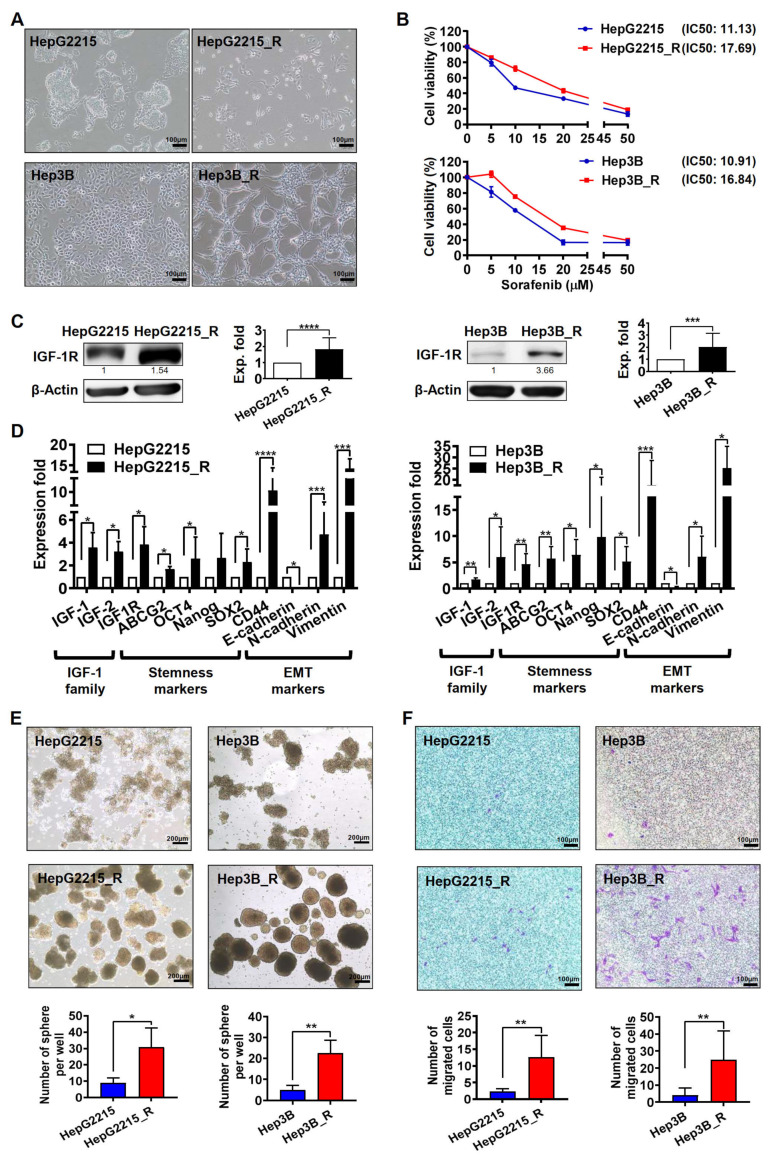
High IGF-1R expression and malignant properties in sorafenib-resistant HCC cell lines. (**A**) Cell morphology of sorafenib-naïve HCC cells (HepG2215 and Hep3B) and sorafenib-resistant HCC cells (HepG2215_R and Hep3B_R). (**B**) Cell viability and IC50 values of sorafenib-naïve HCC cells (HepG2215 and Hep3B) and sorafenib-resistant HCC cells (HepG2215_R and Hep3B_R) under sorafenib treatment (0, 5, 10, 15, 20 and 50 μM). (**C**) Expression of IGF-1R protein in sorafenib-naïve HCC cells (HepG2215 and Hep3B) and sorafenib-resistant HCC cells (HepG2215_R and Hep3B_R). The results of three replicates were shown in Figure S6. (**D**) Gene expression profile of sorafenib-naïve HCC and sorafenib-resistant HCC obtained using real-time RT-qPCR analyses. (**E**) Secondary sphere formation assay. Bar = 100 μm. (**F**) Migration ability assay. Bar = 100 μm. For all quantification, data are the mean ± SD of at least three independent experiments. * *p* < 0.05, ** *p* < 0.01, *** *p* < 0.001, and **** *p* < 0.0001, as obtained from Student’s *t* test.

To examine the differential gene expression profiles of HCC cells with different sorafenib sensitivities, two pairs of HCC cell lines, namely sorafenib-naïve (HepG2215 and Hep3B) and sorafenib-resistant (HepG2215_R and Hep3B_R) HCC cells, were used in this study. In comparison with the sorafenib-naïve HCC cells, the sorafenib-resistant HCC cells exhibited a more mesenchymal cell morphology (Figure 1A), had a higher half-maximal inhibitory concentration (IC50) value under sorafenib treatment (Figure 1B), and had significantly higher IGF-1R protein expression level (Figure 1C). Western blotting, in which 4G10 antibodies were used for detecting the cellular protein tyrosine phosphorylation, revealed that sorafenib effectively suppressed the IGF-1-induced protein tyrosine phosphorylation such as p-IGF-1R, p-PDGFR and p-VEGFR in the sorafenib-naïve HepG2215 and Hep3B cells, but not in their sorafenib-resistant counterparts HepG2215_R and Hep3B_R cells (Supplementary Figure S1A,B). Additionally, the sorafenib-resistant HCC cells exhibited more favorable stemness-related properties, including stemness-related genes (*OCT4*, *NANOG*, *SOX2*) and EMT-related genes (*CD44*, *N-CAD*, *Vimentin*) (Figure 1D), secondary sphere formation ability (Figure 1E), and cell migration ability (Figure 1F). The quantitative analysis in both Figure 1E,F revealed significant results.

### 3.2. Niclosamide Significantly Enhanced the Sorafenib Sensitivity (as Indicated by Cell Viability) and Suppressed the Stemness-Related Properties of Sorafenib-Resistant HCC Cells

**Figure 2:**
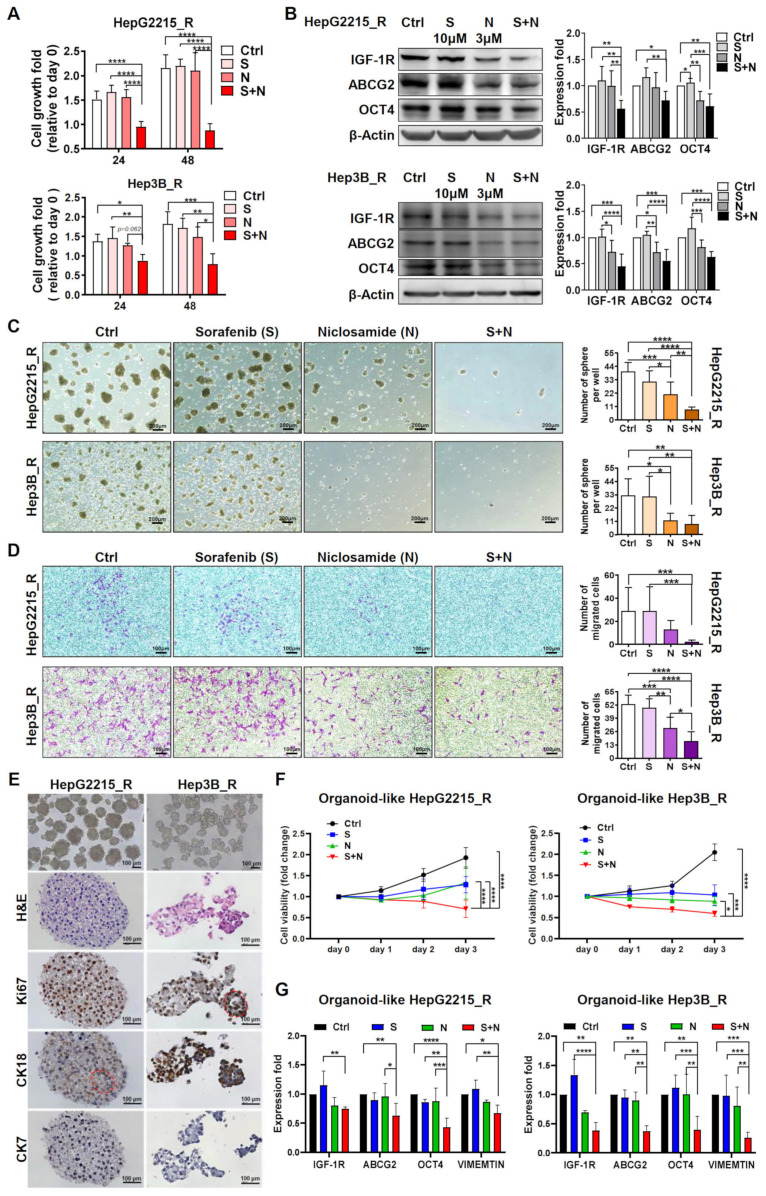
The combination of niclosamide and sorafenib enhances cell death and reduces the activity of stemness-related properties in sorafenib-resistant HCC cells and their organoids. (**A**) Cell viability assay of the sorafenib-resistant HCC cells under different experimental conditions for 24 and 48 h. (**B**) Protein expression in the sorafenib-resistant HCC cells under different experimental conditions for 24 h, including stemness-related markers (IGF-1R, OCT4) and drug resistance markers (ABCG2). The results of three replicates were shown in Figures S7 and S8. (**C**) Secondary sphere formation assay. Bar = 100 μm. (**D**) Migration ability assay. Bar = 100 μm. (**E**) Generation and identification of tumor organoids derived from the sorafenib-resistant HepG2215_R and Hep3B_R cells. The red circle shows the pseudoglandular pattern structure of HCC. Bar = 100 μm. (**F**) Cell viability assay of the sorafenib-resistant HCC organoids under different experimental conditions for 72 h. (**G**) Gene expression of the sorafenib-resistant HCC cells under different experimental conditions for 24 h, including stemness-related markers (*OCT4*), drug resistance (*ABCG2*), and EMT (*IGF-1R* and *VIMENTIN*). Control group (Ctrl), sorafenib alone (S, 10 µM), niclosamide alone (N, 3 µM), and niclosamide plus sorafenib (S + N). For all quantification, data are the mean ± SD of at least three independent experiments. * *p* < 0.05, ** *p* < 0.01, *** *p* < 0.001, and **** *p* < 0.0001, as obtained from one-way ANOVA.

Sorafenib or niclosamide alone was unable to suppress the viability of sorafenib-resistant cells HepG2215_R and Hep3_R (Figure 2). Notably, niclosamide effectively and significantly increased the sorafenib sensitivity of sorafenib-resistant HCC cells by reducing their cell viability (Figure 2A and Supplementary Figure S2A). Further experiments using real-time reverse-transcription quantitative PCR (RT-qPCR) and Western blotting demonstrated that the combination of niclosamide and sorafenib effectively and significantly suppressed both the gene and protein expression levels of pluripotent transcriptional factor OCT4, drug resistance–related ABCG2, and the cell migration–associated IGF-1R in sorafenib-resistant HCC cells (Figure 2B and Supplementary Figure S2B). Additionally, in sorafenib-resistant HCC cells, niclosamide effectively enhanced the suppressive effect of sorafenib on secondary tumor sphere formation (Figure 2C) and cell migration ability (Figure 2D).

### 3.3. The Combination of Niclosamide and Sorafenib Reduced the Cell Viability of Sorafenib-Resistant HCC-Derived Organoids

To mimic the characteristics of solid tumors in HCC, organoids derived from the sorafenib-resistant HepG2215_R and Hep3B_R cell lines were used in this study (Supplementary Figure S3). The sorafenib-resistant HCC–derived organoids were successfully generated, as confirmed by their cluster morphology resembling that of HCC (hematoxylin and eosin [H&E] staining). Further analysis by immunohistochemical staining revealed that the HCC organoids had high proliferation ability (Ki67 staining) and expressed the HCC biomarkers CK18 and CK7 (Figure 2E). Relative to the control group, or sorafenib or niclosamide alone, the combination of niclosamide and sorafenib had a significantly greater suppression effect on cell viability (Figure 2F and Supplementary Figure S4) and on the gene expression of *IGF-1R*, *ABCG2*, *OCT4*, and *VIMENTIN* (Figure 2G) in the sorafenib-resistant HCC organoids. Overall, these results strongly suggest that the combination of niclosamide and sorafenib effectively suppressed the growth of both the sorafenib-resistant HCC cells and their derived HCC organoids.

### 3.4. Niclosamide Effectively Mitigated IGF-1R and OCT4 Expressions as Well as Sorafenib-Induced IGF-1R Phosphorylation in the Sorafenib-Resistant HCC Cells under IGF-1 Treatment

**Figure 3:**
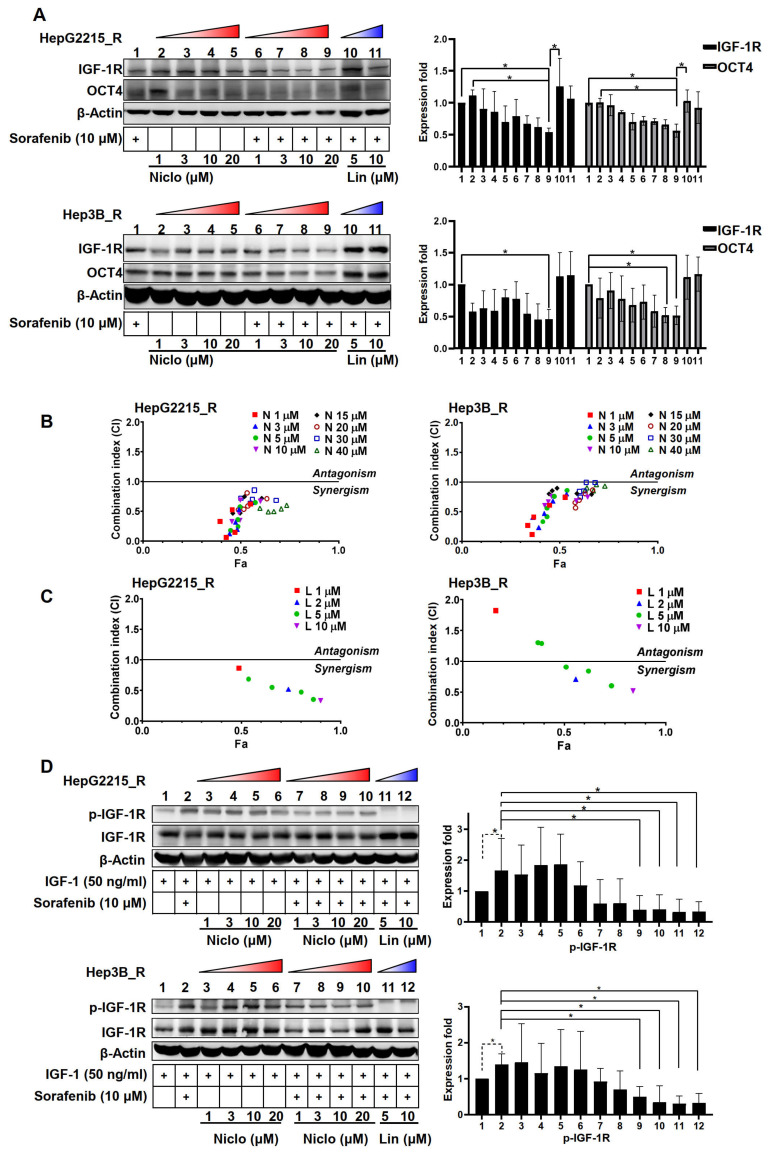
Niclosamide effectively mitigated IGF-1R and OCT4 expression as well as sorafenib-induced IGF-1R phosphorylation in the sorafenib-resistant HCC cells under IGF-1 treatment. (**A**) Effect of niclosamide (Niclo, 1, 3, 10, and 20 μM) or linsitinib (L, 5, 10, and 20 μM) on the expression of IGF-1R and stemness-related protein OCT4 in the sorafenib-resistant HCC cells. The results of three replicates were shown in Figure S9. (**B**) Cell viability assays (WST) of sorafenib-resistant HCC cells treated with niclosamide (1, 3, 5, 10, 15, 20, 30, and 40 µM) with or without sorafenib (0.5, 2.5, 5, 10, and 15 µM) for 24 h. The detailed data was shown in Table S3. (**C**) Cell viability assays (WST) of sorafenib-resistant HCC treated with linsitinib (1, 2, 5, and 10 µM) with or without sorafenib (1, 2.5, 5, and 10 µM) for 24 h. CI values were interpreted as follows: CI > 1, antagonistic effect; CI = 1, additive effect; and CI < 1, synergistic effect. The detailed data was shown in Table S3. (**D**) Effect of niclosamide (Niclo, 1, 3, 10, and 20 μM) or linsitinib (Lin, 5, 10, and 20 μM) on IGF-1/sorafenib-induced IGF-1R phosphorylation of the sorafenib-resistant HCC cells. The results of three replicates were shown in Figure S11. * *p* < 0.05 as obtained from one-way ANOVA.

The effect of niclosamide on the sorafenib-mediated expression of IGF-1R and OCT4 in the sorafenib-resistant HepG2215_R and Hep3B_R cell lines was examined (Figure 3A, Lanes 6–9 vs. Lane 1; Supplementary Figure S5). The protein expression levels of IGF-1R were similar to those of OCT4 in the sorafenib-resistant HCC cells. Linsitinib markedly increased both IGF-1R and OCT4 in Hep3B_R in comparison with the control group (Lanes 10 and 11 vs. Lane 1). The linsinitib alone fails to reduce IGF-1R expression in sorafenib-resistant HCC cells (Supplementary Figure S10). These results highlight the significant suppressive effect of niclosamide on the expression of IGF-1R and OCT4 in sorafenib-resistant HCC cells.

The effect of the combination of niclosamide and sorafenib on sorafenib-resistant HCC cells was subsequently examined by considering the CI values. We found that niclosamide significantly increased sorafenib sensitivity in a dose-dependent manner. Niclosamide and sorafenib had a synergistic effect on the proliferative suppression of the two sorafenib-resistant cell lines (Figure 3B). There is only a limited effect of linsitinib on sorafenib sensitivity when compared with niclosamide (Figure 3C). The IC50 of linsitinib (HepG2215_R, IC_50_:43.95 μM; Hep3B_R, IC_50_:30.80 μM) and niclosamide (HepG2215_R IC_50_:15.08 μM; Hep3B_R, 17.50 μM) were shown in Supplementary Table S3. These results demonstrate that the combination of niclosamide and sorafenib had a synergistic effect in the treatment of the sorafenib-resistant HCC cells.

To further examine the potential suppressive effect of niclosamide on the IGF-1R protein phosphorylation in sorafenib-resistant HCC cells, the sorafenib-resistant Hep2215_R and Hep3B_R HCC cells were treated with IGF-1 (50 ng/mL) with or without the presence of sorafenib (10 µM). The protein levels of p-IGF-1R and IGF-1R were detected using Western blot analysis. As indicated in Figure 3D, sorafenib significantly increased the IGF-1-induced p-IGF-1R levels both in the HepG2215_R and Hep3B_R cells (Lane 2 vs. Lane 1). Without sorafenib, the niclosamide alone (1, 3, 10, or 20 µM) only showed a weak suppressive effect on the IGF-1-induced p-IGF-1R intensity (Lanes 3–6 vs. Lane 1). Notably, a combination of niclosamide and sorafenib treatment drastically and significantly suppressed the sorafenib-induced p-IGF-1R intensity of sorafenib-resistant HCC cells under IGF-1 treatment (Lanes 7–10 vs. Lane 2). The linsitinib, an IGF-1R phosphorylation inhibitor, served as a positive control in p-IGF-1R suppression (Lanes 11–12). These results demonstrate that the niclosamide effectively suppressed the sorafenib-induced IGF-1R phosphorylation in the sorafenib-resistant HCC cells under IGF-1 treatment.

### 3.5. The Combination Niclosamide and Sorafenib Suppressed the Sorafenib-Induced Glycolysis-Related Genes in Sorafenib-Resistant HCC Cells and Their Organoids

**Figure 4:**
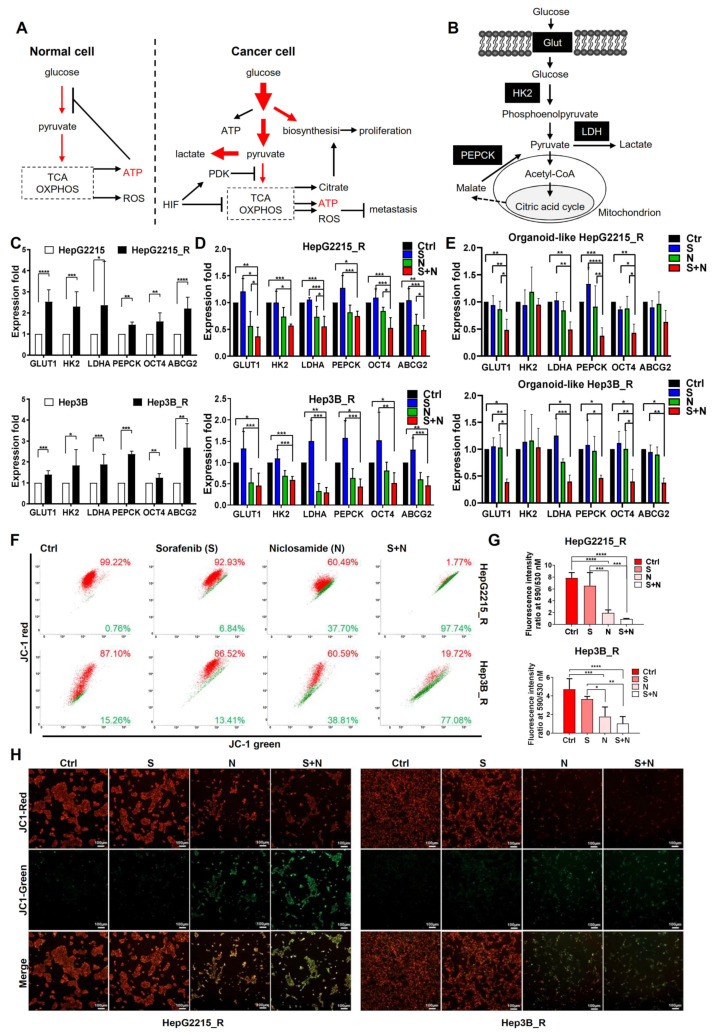
Niclosamide enhanced the ability of sorafenib by decreasing glycolysis and mitochondrial membrane potential in sorafenib-resistant HCC cells. (**A**) Schematic of glucose metabolism in normal cells and cancer cells under normoxia. (**B**) Representative enzymes in glycolysis and oxidative phosphorylation. Glut, glucose transporter; HK2, hexokinase 2; LDH, lactic dehydrogenase; PEPPCK, phosphoenolpyruvate carboxykinase. (**C**) Gene expression levels of glycolysis-related enzymes in both sorafenib-naïve and sorafenib-resistant HCC cells (**D**) or their organoids (**E**) under different experimental conditions for 24 h were shown. Control group (Ctrl), sorafenib alone (S, 10 µM), niclosamide alone (N, 3 µM), and niclosamide plus sorafenib (S + N). Real-time RT-qPCR analyses. (**F**) JC-1 staining levels of sorafenib-resistant HCC cells (HepG2215_R and Hep3B_R) under different experimental conditions for 48 h. Control group (Ctrl), sorafenib alone (S, 10 µM), niclosamide alone (N, 3 µM), and niclosamide plus sorafenib (S + N). Flow cytometry dot plot analysis for the gating of JC1 (red)-aggregates and JC1 (green)-monomer populations. (**G**) Fluorescence intensity ratio at 590 nm/530 nm and (**H**) the cell images of (**F**). * *p* < 0.05, ** *p* < 0.01, *** *p* < 0.001, and **** *p* < 0.0001, as obtained from one-way ANOVA.

Cancer cells are known to increase glucose uptake and glycolytic rates, referred to as aerobic glycolysis or the Warburg effect (Figure 4A). Figure 4B presents the metabolic schematics with representative enzymes in both glycolysis and oxidative phosphorylation. We found that, in comparison with the naïve cells, the sorafenib-resistant HCC cells had higher expression levels of genes associated with glycolysis (*GLUT1*, *HK2*, and *LDHA*) and stemness (*OCT4* and *ABCG2*) (Figure 4C). We subsequently examined the effect of a combination of niclosamide and sorafenib treatment on gene expression. Relative to the control group or the sorafenib or niclosamide alone, the combination of niclosamide and sorafenib had a significantly stronger suppression effect on the expression of glycolysis-related and stemness-related genes in both the sorafenib-resistant HCC cells (Figure 4D) and the HCC organoids (Figure 4E). These results highlight that the combination of niclosamide and sorafenib reduced both the glycolysis-related and stemness-related gene expression in the sorafenib-resistant HCC cells.

### 3.6. Niclosamide Enhanced the Ability of Sorafenib to Reduce Mitochondrial Membrane Potential in Sorafenib-Resistant HCC Cells

To examine the effect of niclosamide on the ability of sorafenib to reduce the mitochondrial membrane potential of sorafenib-resistant HCC cells, the JC-1 fluorescent cationic probe for mitochondrial membrane potential (ΔΨm)—was employed. JC-1 aggregates in mitochondria emit red fluorescence (high membrane potential), whereas JC-1 monomers emit green fluorescence in the case of mitochondrial membrane depolarization (low membrane potential). Dot plot analysis revealed that niclosamide markedly enhanced the ability of sorafenib to reduce mitochondrial membrane potential in sorafenib-resistant HCCs (Figure 4F). Relative to the control group (dimethyl sulfoxide; DMSO), the sorafenib or niclosamide treatment alone had only a weak to moderate effect on the decrease in mitochondria membrane potential (JC-1 aggregates, red fluorescence, sorafenib vs. control: 92.93% vs. 99.22% for HepG2215_R, and 86.52% vs. 87.10% for Hep3B_R; niclosamide vs. control: 60.49% vs. 99.22% for HepG2215_R, and 60.59% vs. 87.10% for Hep3B_R, respectively). The combination of niclosamide and sorafenib effectively and significantly reduced the JC-1 aggregates (JC-1 red) and increased the percentage of JC-1 monomers (JC-1 green) (Figure 4F). We observed a significant decrease in the fluorescence ratio of JC-1 red/JC-1 green (590/530 nm) in the sorafenib-resistant HepG2215_R and Hep3B_R cells (Figure 4G). These findings were further verified through fluorescence images. Relative to the control or the sorafenib-only groups, the niclosamide-only or sorafenib-plus-niclosamide groups exhibited a considerable decrease in red fluorescence (an aggregated form of JC-1) and an increase in green intensity (monomer form of JC-1) in sorafenib-resistant HCCs (Figure 4H).

### 3.7. The Combination of Niclosamide and Sorafenib Suppressed Tumor Growth by Increasing the Cell Apoptosis in Sorafenib-Resistant HCC Cells In Vivo

**Figure 5:**
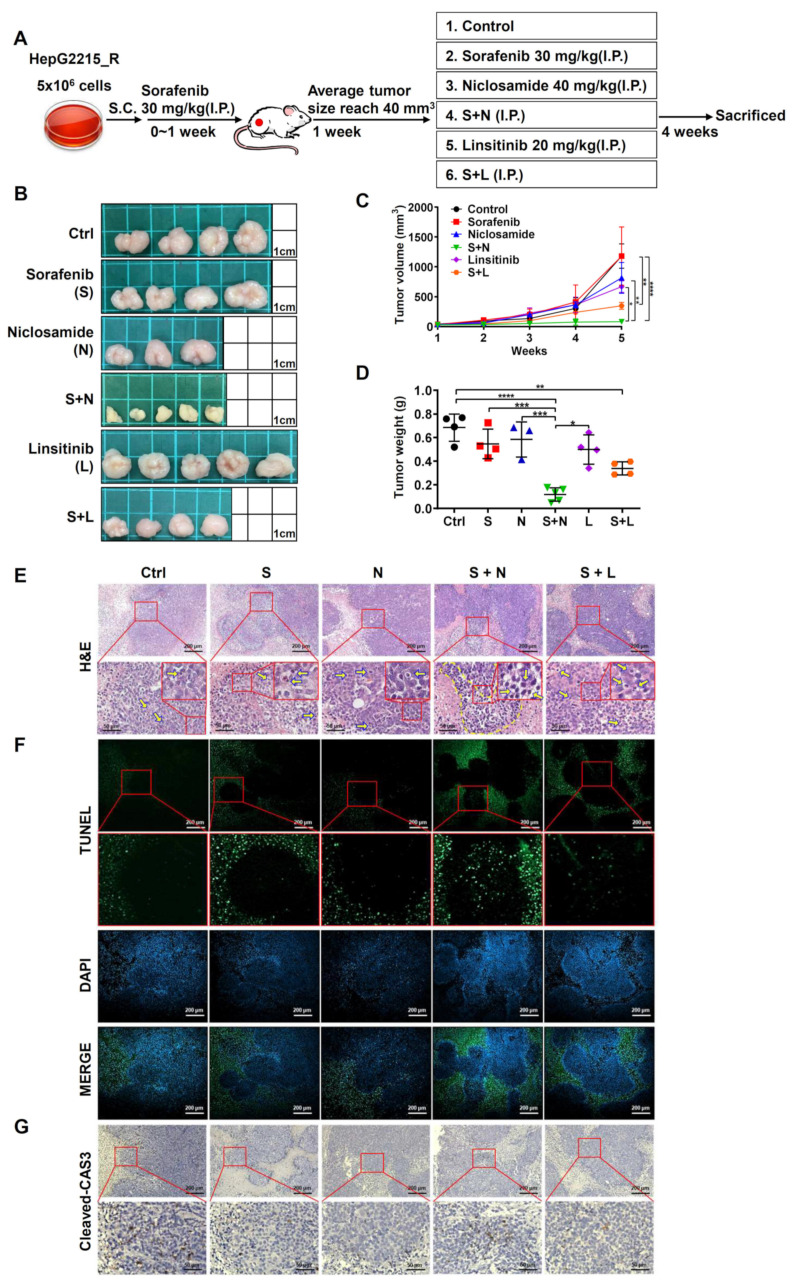
Combination treatment with niclosamide and sorafenib suppressed tumor growth and increased cell apoptosis in sorafenib-resistant xenograft tumors. (**A**) Schematic diagram of the animal model experimental design. (**B**) Tumor tissues of the different experimental conditions. Control group (Ctrl), sorafenib alone (S, 30 mg/kg), niclosamide alone (N, 40 mg/kg), niclosamide plus sorafenib (S + N), linsitinib (L, 20 mg/kg), and linsitinib plus sorafenib (S + L). (**C**) Tumor volume analysis. (**D**) Tumor weight analysis. (**E**) H&E staining of the sorafenib-resistant xenograft tumor tissues of different treatments. Control group (Ctrl), sorafenib alone (S, 30 mg/kg), niclosamide alone (N, 40 mg/kg), niclosamide plus sorafenib (S + N), linsitinib (L, 20 mg/kg), and linsitinib plus sorafenib (S + L). (**F**) TUNEL immunostaining (in green) and DAPI staining (in blue) of (**G**). Expression levels and cellular localization of cleaved caspase 3 protein (cleaved-CAS3) in xenograft of sorafenib-resistant HepG2215_R tumor tissues are shown. Scale bar, 100 µm. * *p* < 0.05, ** *p* < 0.01, *** *p* < 0.001, and **** *p* < 0.0001, as obtained from one-way ANOVA.

To examine whether the combination of either niclosamide or linsitinib (p-IGF1R inhibitor) with sorafenib can suppress tumor growth in sorafenib-resistant HCC cells in vivo, tumor-bearing NOD-SCID mice were treated with sorafenib (30 mg/kg) for 1 week. Subsequently, the sorafenib-treated mice were divided into six differential treatment groups for an additional 4 weeks: control (DMSO), sorafenib alone (30 mg/kg), niclosamide alone (40 mg/kg), niclosamide plus sorafenib, linsitinib alone (20 mg/kg), and linsitinib plus sorafenib (Figure 5A); all treatments were administered intraperitoneally. In line with the in vitro cell assay results, the single use of either sorafenib, niclosamide, or linsitinib only resulted in a weak effect; by contrast, the combination of sorafenib and niclosamide significantly decreased both the tumor volume (Figure 5B,C) and the tumor weight (Figure 5D) of sorafenib-resistant HCC cells in vivo. The combination of linsitinib and sorafenib only had a mild suppressive effect on tumor growth (Figure 5B–D). The results of triple animal experiments were shown in Figure S12.

To examine how niclosamide treatment increases the sorafenib sensitivity of the HCC cells, xenograft sorafenib-resistant HCC tumor tissues were collected for immunohistochemical staining. In comparison with the control, sorafenib alone, or niclosamide alone groups, the combination treatment with niclosamide and sorafenib markedly increased the cell apoptosis in sorafenib-resistant HCC tumor tissues. The combination of linsitinib and sorafenib only had a mild effect (Figure 5E,F; H&E staining and TUNEL assay). These results were further verified through immunohistochemical staining against the cleaved caspase 3 protein (Figure 5G). These results strongly demonstrate the role of niclosamide in enhancing the sorafenib sensitivity of sorafenib-resistant HCC cells in vivo. The underlying mechanism may involve an increase in the tumor cell apoptosis of sorafenib-resistant HCC cells.

## 4. Discussion

Drug repurposing has a shorter approval process than new drug development. Niclosamide is an FDA-approved antihelminthic drug that suppresses glycemia [9]; this drug has also been reported to inhibit cancer malignancy through various mechanisms, including Wnt/β-catenin [24], mTORC1 [25], STAT3 [26], NFκB [27], S100A4 signaling [28], and other signaling pathways [29,30]. The anticancer activities of niclosamide have proven beneficial in colon [31], prostate [20], ovary [30], and breast cancers [26]; HCC [32]; and many other cancer types [33]. These results suggest that niclosamide can be repurposed for novel cancer treatments beyond helminthiasis [18]. In this paper, advanced from the previous studies, we demonstrated the repurposing of niclosamide to increase the sorafenib sensitivity of sorafenib-resistant HCC cells by using both cell lines (two-dimensional culture) and an organoid system (three-dimensional culture). The combination of niclosamide and sorafenib provides a practical clinical strategy for increasing the sorafenib sensitivity of HCC cells, in addition to contributing to the development of an effective therapeutic strategy for patients with sorafenib-refractory HCC.

Relevant clinical trials on niclosamide for cancer treatment have examined (1) the combination of enzalutamide and niclosamide in treating patients with recurrent or metastatic castration-resistant prostate cancer (NCT02532114 and NCT03123978, Phase 1); (2) the combination of abiraterone acetate, niclosamide, and prednisone in treating patients with hormone-resistant prostate cancer (NCT02807805, Phase 2); (3) the safety and efficacy of niclosamide tablets in patients with metastatic colorectal cancer progressing after therapy (NCT02519582, Phase 2); and (4) the use of niclosamide in pediatric patients with relapsed and refractory acute myeloid leukemia (NCT05188170, Phase 1).

Medical treatment of HCC has been hampered by the limited availability of effective systemic treatment options; moreover, de novo or treatment-induced resistance to these drugs is common. To date, multitargeted tyrosine kinase inhibitors such as sorafenib (Nexavar), regorafenib (Stivarga), lenvatinib (Lenvima), and cabozantinib (Carbometyx) have been approved for HCC treatment. Sorafenib is the first targeted systemic therapy approved for unresectable HCC. Over the past 20 years, sorafenib has become known for offering considerable survival benefits in advanced HCC [34]. Several mechanisms that contribute to acquired refractoriness to sorafenib have been reported [13,35], with examples including the PI3K/AKT [36,37], Raf/Mek/ERK [38], and Jak/Stat3 [39,40] signaling pathways; activation of hypoxia-inducible pathways [41]; epithelial–mesenchymal transition [42]; enrichment of tumor-initiating cell population [43]; microenvironmental and metabolic derangement [44]; and autophagy [45]. Despite considerable research on this drug, sorafenib resistance in patients with HCC remains a large concern, with few answers offered in the literature.

Metabolic alteration has been proposed as one mechanism through which HCC cells acquire resistance to sorafenib [46,47,48], with aerobic glycolysis (the Warburg effect) being a well-known malignant phenotype [27,32]. Through a reduction in the cell dependence on oxygen and the mitochondria-derived reactive oxygen species, glycolysis regulates oxidant-induced senescence and apoptosis, thereby protecting the cancer cells in tissues [24,25]. In line with the aforementioned findings, a recent report indicated that drug treatment increases the glycolysis ability of liver cancer cells [49]. These observations highlight the potential of glycolysis blockade, through the action of niclosamide, for example, for killing cancer cells.

In addition, niclosamide was reported to serve as a mitochondrial uncoupler to inhibit the anabolic effect of aerobic glycolysis in colon cancer cells [50]. Niclosamide has also been found to uncouple mitochondria, reduce liver steatosis, and increase insulin sensitivity [51]. Notably, the combination treatment with niclosamide and paclitaxel was more efficacious than paclitaxel alone and was noted to increase paclitaxel sensitivity in paclitaxel-resistant esophageal cancer cells [52]. These results emphasize that niclosamide may serve as a concomitant agent for treating drug-resistant cancer.

We adopted an organoid model in this study. When compared with a 2D cell culture system, an HCC organoid model more accurately represents the tumor tissue pathology. Researchers have conducted comparisons of 2D- and 3D-culture models of both cancer cell lines and patient-derived xenografts as a drug testing platform in breast cancer [12,14,15], lung cancer [11], endometrial cancer [9], and in the coculture of HeLa/ovarian cancer or HeLa/human umbilical vein endothelial cells [18]. These reports have confirmed the advantages of using 3D-culture models for drug screening; such models increase the predictability of in vivo drug response. In this study, we observed the pseudoglandular pattern structure of HCC in both the Hep2215_R and Hep3B_R-derived organoids. Furthermore, the combination of sorafenib and niclosamide had significant suppressive effects on cell viability and gene expression (*IGF-1R*, *OCT4*, *ABCG2*, *VIMENTIN*, and glycolytic genes). Moreover, the organoid model provided an effective monitoring system for examining the effect of certain drugs on tumor size over time.

High expression of IGF-1R is closely associated with cancer stemness properties [53,54] and sorafenib resistance [23] in HCC. IGF-1/IGF-1R signaling has been widely examined for its role in cancer progression through the regulation of cancer-stemness markers as well as EMT markers [23,53,54,55,56,57]. We previously demonstrated that IGF-1R activation increases the activity of stemness-related properties and sorafenib-refractoriness in HCC, thereby contributing to early tumor recurrence [23,53]. We found that the tumor area had a higher phosphorylation level of IGF-1R (p-IGF-1R) than the adjacent nontumor area [53]. Moreover, the IGF-1/2 stimulation resulted in the nuclear translocation of YAP, helping to enhance sorafenib and regorafenib resistance in HCC [23,58]. Similarly, Lippolis et al. observed IGF-1 antagonized regorafenib-mediated cell viability, migration-, and invasion ability with IGF-1 treatment [59]. One phase I clinical trial of linsitinib, a dual inhibitor of IGF-1R and insulin receptors, was terminated because of safety concerns (NCT01101906); however, other phase 1 clinical trials involving combination anti-IGF-1R therapy and sorafenib have reported no safety concerns (BIIB022-NCT00956436 and AVE1642-NCT00791544).

**Figure 6:**
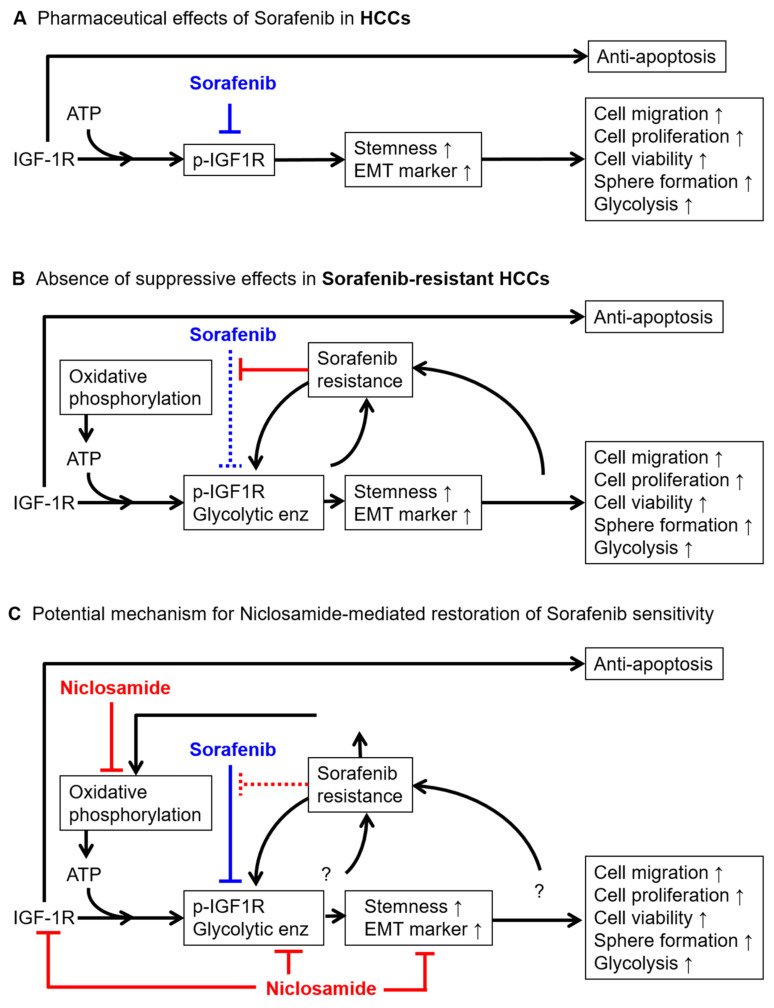
Model for potential niclosamide-mediated restoration of sorafenib sensitivity in sorafenib-resistant HCC cells. (**A**) Pharmaceutical effects of sorafenib in HCC cells. (**B**) Absence of suppressive effects in sorafenib-resistant HCC cells. (**C**) Potential mechanism for niclosamide-mediated restoration of sorafenib sensitivity in sorafenib-resistant HCC cells.

Combination therapy is increasingly being employed in HCC trials [14,15]. In this study, building on previous research, we demonstrated the effect of niclosamide in enhancing HCC sensitivity to sorafenib, in particular through IGF-1/IGF-1R activation, stemness regulation, and metabolic changes. Our previous studies have demonstrated that IGF-1R activation can prevent cell apoptosis and increase the ability of stemness- and EMT-related properties [23,53]. In sorafenib-naïve HCC cells, sorafenib effectively suppressed the IGF-1-induced p-IGF-1R activation (Figure 6A). However, sorafenib-resistant HCC cells had higher expression levels of IGF-1R/p-IGF-1R, the stronger activity of stemness- and EMT-related properties, higher glycolytic enzymes, and greater mitochondria activity (Figure 6B). The present results demonstrated that niclosamide significantly decreases the stemness-related and EMT-related properties (Figure 1 and Figure 2), downregulates IGF-1R and p-IGF-1R (Figure 3), inhibits the expression of glycolytic enzymes and mitochondria membrane potential (Figure 4), and suppresses the cell viability in vitro and in vivo (Figure 2 and Figure 5). These results suggest that niclosamide mediates the restoration of sorafenib sensitivity through the suppression of IGF-1R/stemness and metabolic changes in glycolytic enzymes and mitochondria membrane potential (oxidative phosphorylation), and the stemness-/EMT properties (Figure 6C).

## 5. Conclusions

The IGF-1R, stemness-related properties, and metabolism play a key role in HCC sorafenib resistance. In this study, we demonstrated that niclosamide revitalizes sorafenib through the regulation of IGF-1R/stemness and metabolic changes in HCC using cells/organoids and animal models in vitro and in vivo. The present findings may inform the future development of a practical clinical strategy for repurposing niclosamide for overcoming sorafenib resistance in HCC.

## Data Availability

The data presented in this study are available on reasonable request to the corresponding author.

## Supplementary Materials

The following are available online at https://www.mdpi.com/article/10.3390/cancers15030931/s1, Figure S1: Receptor tyrosine kinase is stably expressed after sorafenib treatment in sorafenib-resistant HCC cells, Figure S2: The combination of niclosamide and sorafenib enhances cell death and reduces the activity of stemness-related properties in sorafenib-resistant HCC cells, Figure S3: Cultivation of organoid-like sorafenib-resistant HCC, Figure S4: The combination of niclosamide and sorafenib suppresses the development of organoid-like HCC, Figure S5: Combination treatment with low-dose niclosamide attenuates sorafenib-induced elevation of IGF-1R and p-IGF-1R elevation in sorafenib-resistant HCC cell lines, Figure S6: The full membrane of western blot results and densitometry readings/intensity ratio for each band of Figure 1C. The protein expression levels of IGF-1R in sorafenib-naïve HCC cells (HepG2215 and Hep3B) and sorafenib-resistant HCC cells (HepG2215_R and Hep3B_R) were shown. The relative quantification was normalized to the corresponding β-Actin, Figure S7: The triple repeats of western blot results and densitometry readings/intensity ratio of each band of main Figure 2B. The protein expression levels of IGF-1R, ABCG2 and OCT4 in sorafenib-resistant HCC cells (HepG2215_R and Hep3B_R) were shown. The relative quantification was normalized to the corresponding β-Actin, Figure S8: The full membrane of western blot results and densitometry readings/intensity ratio for each band of Figure 2B. The protein expression levels of IGF-1R, ABCG2 and OCT4 in sorafenib-resistant HCC cells (HepG2215_R and Hep3B_R) were shown. The relative quantification was normalized to the corresponding β-Actin, Figure S9: The full membrane and triple repeats of western blot analysis and densitometry readings/intensity ratio for each band of main Figure 3A. The protein expression levels of IGF-1R and OCT4 in sorafenib-resistant HCC cells (HepG2215_R and Hep3B_R) were shown. The relative quantification was normalized to the corresponding β-Actin, Figure S10: Linsinitib alone was unable to decrease the IGF-1R expression levels of sorafenib-resistant HCC cells in 24 h treatment. The protein expression levels of IGF-1R in sorafenib-resistant HCC cells (HepG2215_R and Hep3B_R). The relative quantification was normalized to the corresponding GAPDH, Figure S11: The full membrane and triple repeats of western blot analysis and densitometry readings/intensity ratio for each band of main Figure 3D. The protein expression levels of p-IGF-1R in sorafenib-resistant HCC cells (HepG2215_R and Hep3B_R) were shown. The relative quantification was normalized to the IGF-1R then corresponding β-Actin, Figure S12: The three repeats of animal experiments of Figure 5. (A) The size of tumor tissues under different experimental conditions was shown. Control group (Ctrl), sorafenib alone (S, 30 mg/kg), niclosamide alone (N, 40 mg/kg), niclosamide plus sorafenib (S + N), linsitinib (L, 20 mg/kg), and linsitinib plus sorafenib (S + L). (B) Tumor volume analysis. (C) Tumor weight analysis. * *p* < 0.05, ** *p* < 0.01, *** *p* < 0.001, and **** *p* < 0.0001, as obtained from one-way ANOVA, Table S1: The list of real-time PCR primers and their product size, including *IGF-1R, IGF-1, IGF-2, VIMENTIN, N-CAD, E-CAD, ABCG2, OCT4, NANOG, SOX2, CD44, β-2M, GLUT1, LDHA, PEPCK, HK2*, Table S2: The list of antibodies used in this study. Table S3. The detailed data of Figure 3B,C. The table shows the raw data used when counting combination index.
