# Supplementary material for: Niclosamide Revitalizes Sorafenib through Insulin-like Growth Factor 1 Receptor (IGF-1R)/Stemness and Metabolic Changes in Hepatocellular Carcinoma
Source: Cancers (Basel). 2023 Feb 1;15(3):931. doi: 10.3390/cancers15030931 (PMC9913083; doi:10.3390/cancers15030931)
Supplement: Supplementary file 1 [file cancers-15-00931-s001.zip › cancers-2143598-supplementary/20230201_1720am_cancers-2143598-supplementary.pdf]

Supplemental information

1. Supplemental Figures

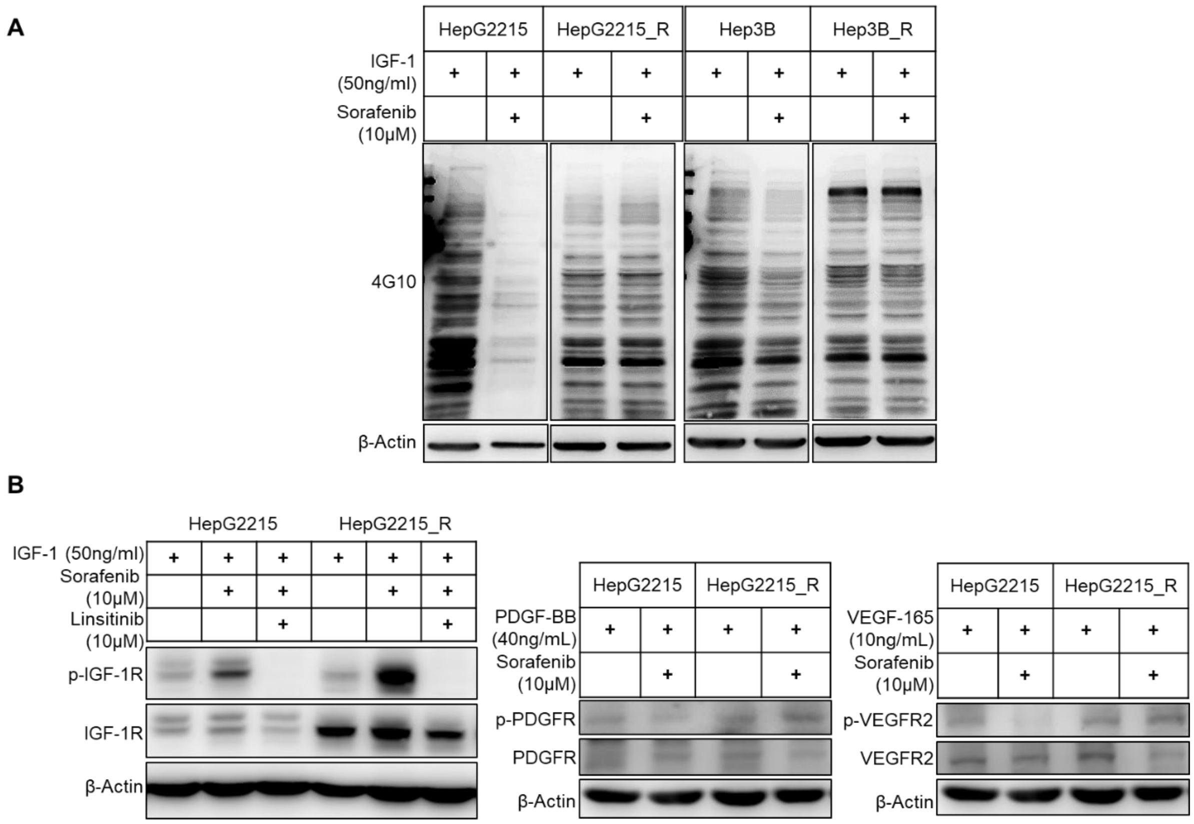

Supplementary Figure S1. Receptor tyrosine kinase is stably expressed after sorafenib treatment in sorafenib-resistant HCC cells. **(A)** Expression of receptor tyrosine kinase in sorafenib-naïve HCC (HepG2215 and Hep3B) and sorafenib-resistant HCC (HepG2215_R and Hep3B_R) cells. **(B)** Expression of p-IGF-1R, p-PDGFR and p-VEGFR in sorafenib-naïve HCC (HepG2215) and sorafenib-resistant HCC (HepG2215_R) cells.

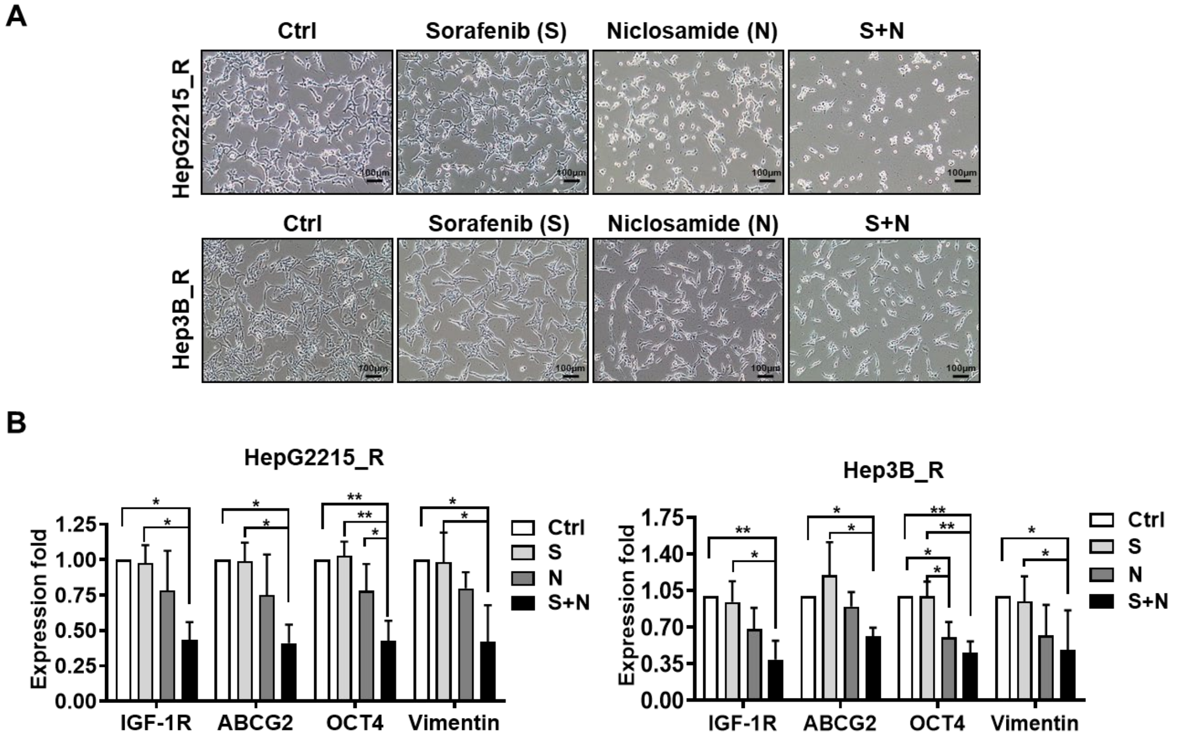

Supplementary Figure S2. The combination of niclosamide and sorafenib enhances cell death and reduces the activity of stemness-related properties in sorafenib-resistant HCC cells. **(A)** Cell images of sorafenib-resistant HepG2215_R and Hep3B_R cells under various conditions: control (Ctrl), sorafenib alone (S, 10 μM), niclosamide alone (N, 3 μM), and niclosamide plus sorafenib (S+N) for 24 h treatment. **(B)** Gene expression in the sorafenib-resistant HCC cells under different experimental conditions for 24 h, including stemness-related markers (*IGF-1R*, *OCT4*, *CD44*), drug resistance (*ABCG2*), and EMT (*Vimentin*). *$p < 0.05$, **$p < 0.01$, ***$p < 0.001$, and ****$p < 0.0001$, as obtained from one-way ANOVA.

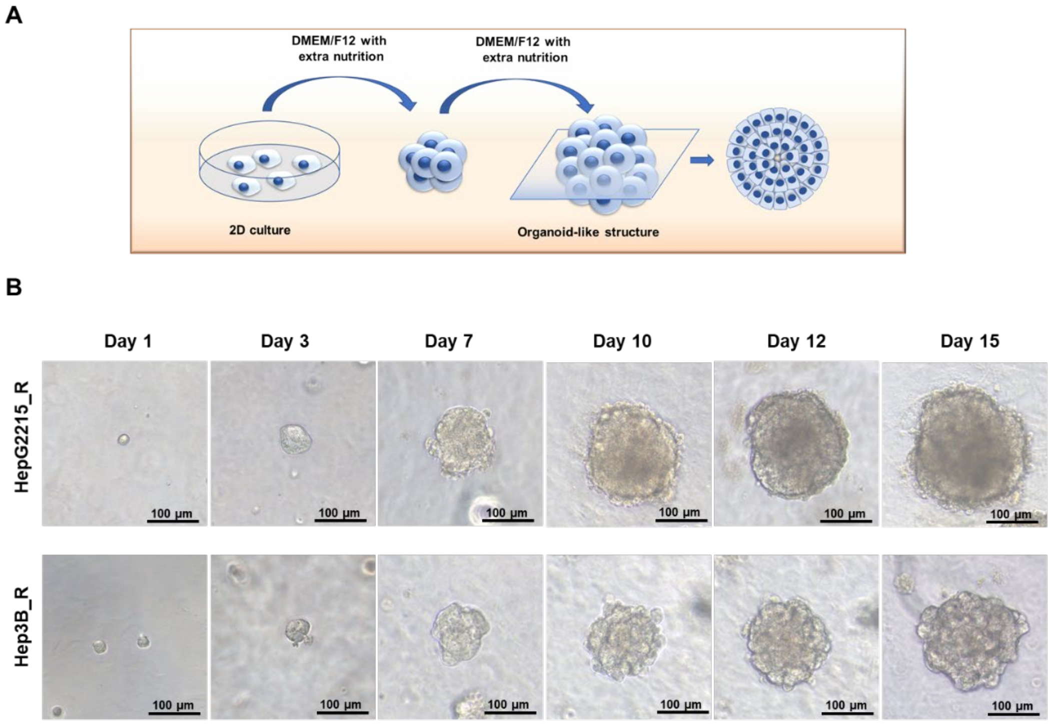

Supplementary Figure S3. Cultivation of organoid-like sorafenib-resistant HCC. **(A)** Diagram of organoid culture protocol**. (B)** Morphology of organoids in culture. Bar = 100 µm.

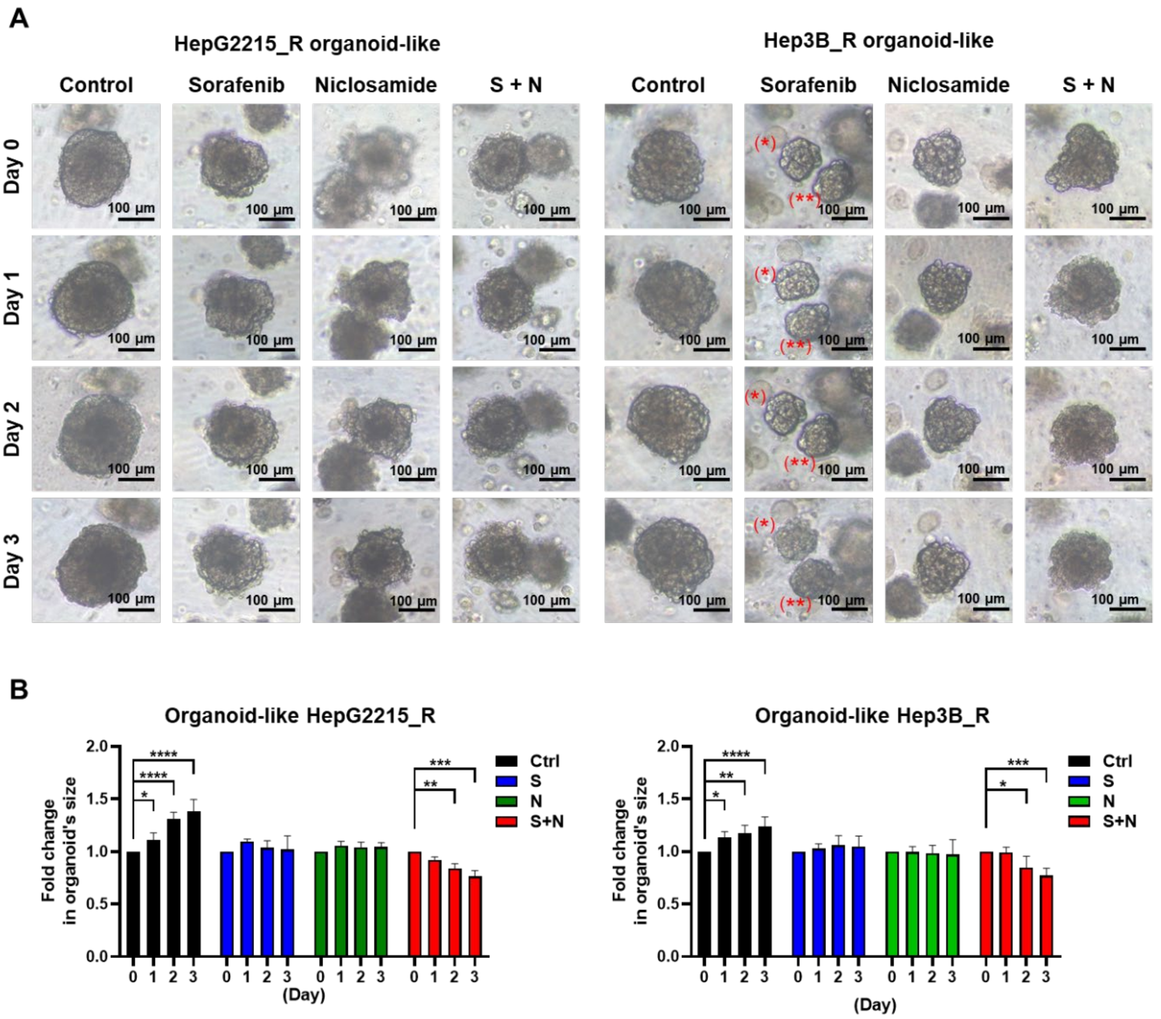

Supplementary Figure S4. The combination of niclosamide and sorafenib suppresses the development of organoid-like HCC. **(A)** Bright field image of organoids treated with sorafenib (10 μM) and niclosamide (3 μM) alone or in combination. **(B)** Changes in the size of organoids treated with sorafenib (10 μM) and niclosamide (3 μM) alone or in combination. Organoid sizes were calculated using Fiji software. Scale bar, 100 μm. Data are the mean ± SD of $n = 5$. *$p < 0.05$, **$p < 0.01$, ***$p < 0.001$, and ****$p < 0.0001$, as obtained from one-way ANOVA.

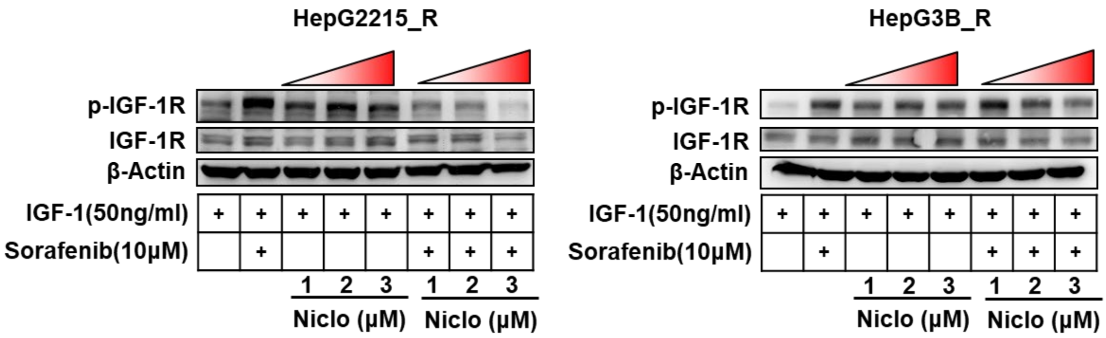

Supplementary Figure S5. Combination treatment with low-dose niclosamide attenuates sorafenib-induced elevation of IGF-1R and p-IGF-1R elevation in sorafenib-resistant HCC cell lines. Effect of niclosamide (Niclo, 1, 2, and 3 μM) on IGF-1/sorafenib-induced IGF-1R phosphorylation in the sorafenib-resistant HCC cells.

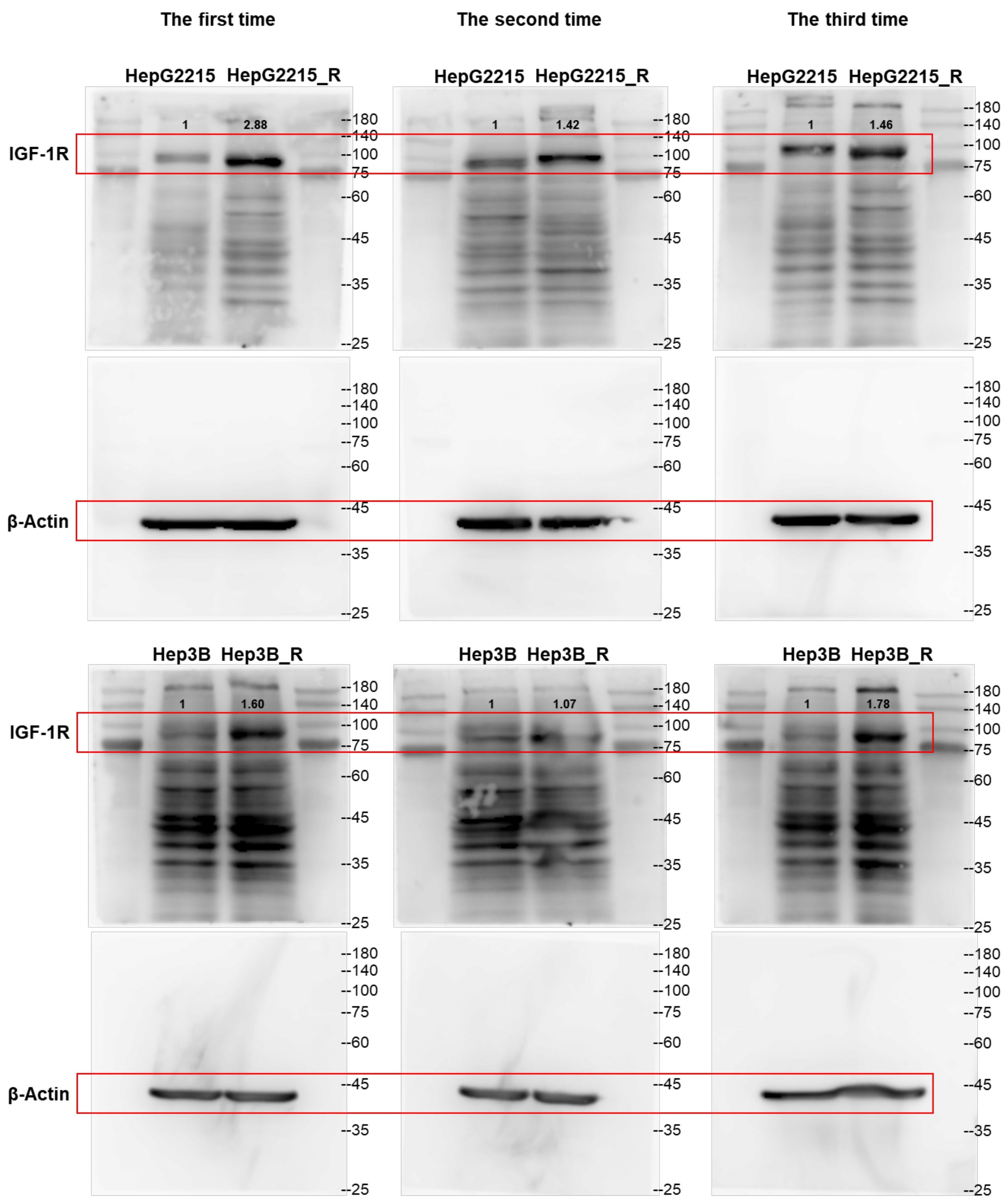

Supplementary Figure S6. The full membrane of western blot results and densitometry readings/intensity ratio for each band of Figure 1C. The protein expression levels of IGF-1R in sorafenib-naïve HCC cells (HepG2215 and Hep3B) and sorafenib-resistant HCC cells (HepG2215_R and Hep3B_R) were shown. The relative quantification was normalized to the corresponding β-Actin.

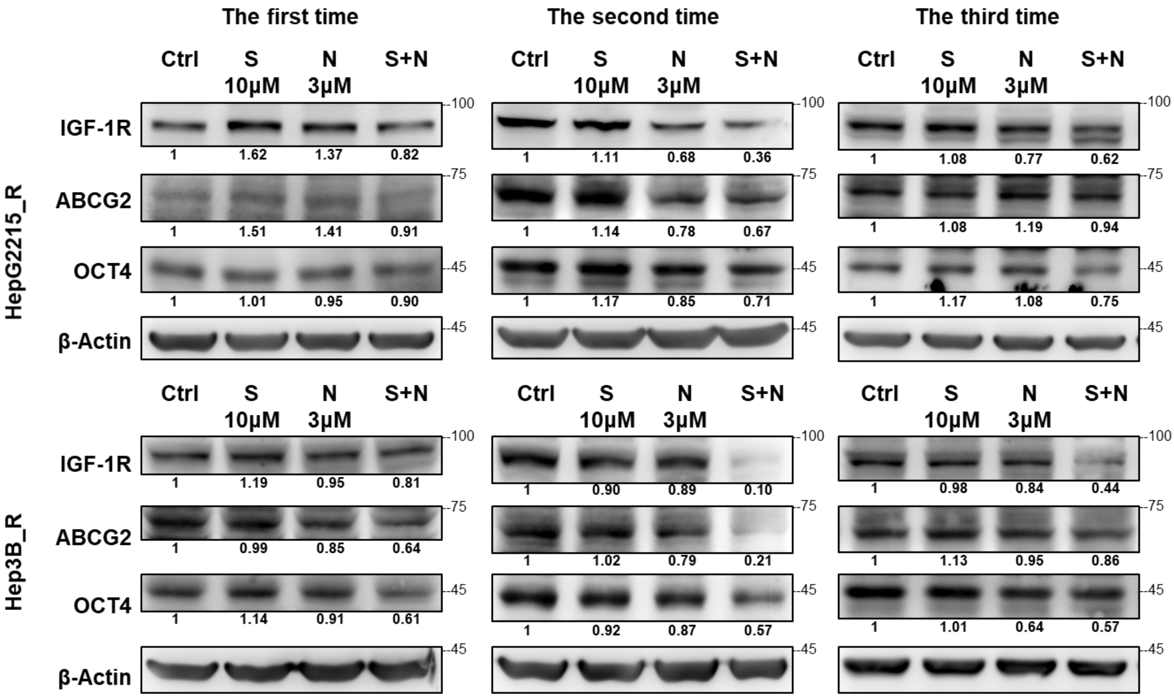

Supplementary Figure S7. The triple repeats of western blot results and densitometry readings/intensity ratio of each band of main Figure 2B. The protein expression levels of IGF-1R, ABCG2 and OCT4 in sorafenib-resistant HCC cells (HepG2215_R and Hep3B_R) were shown. The relative quantification was normalized to the corresponding β-Actin.

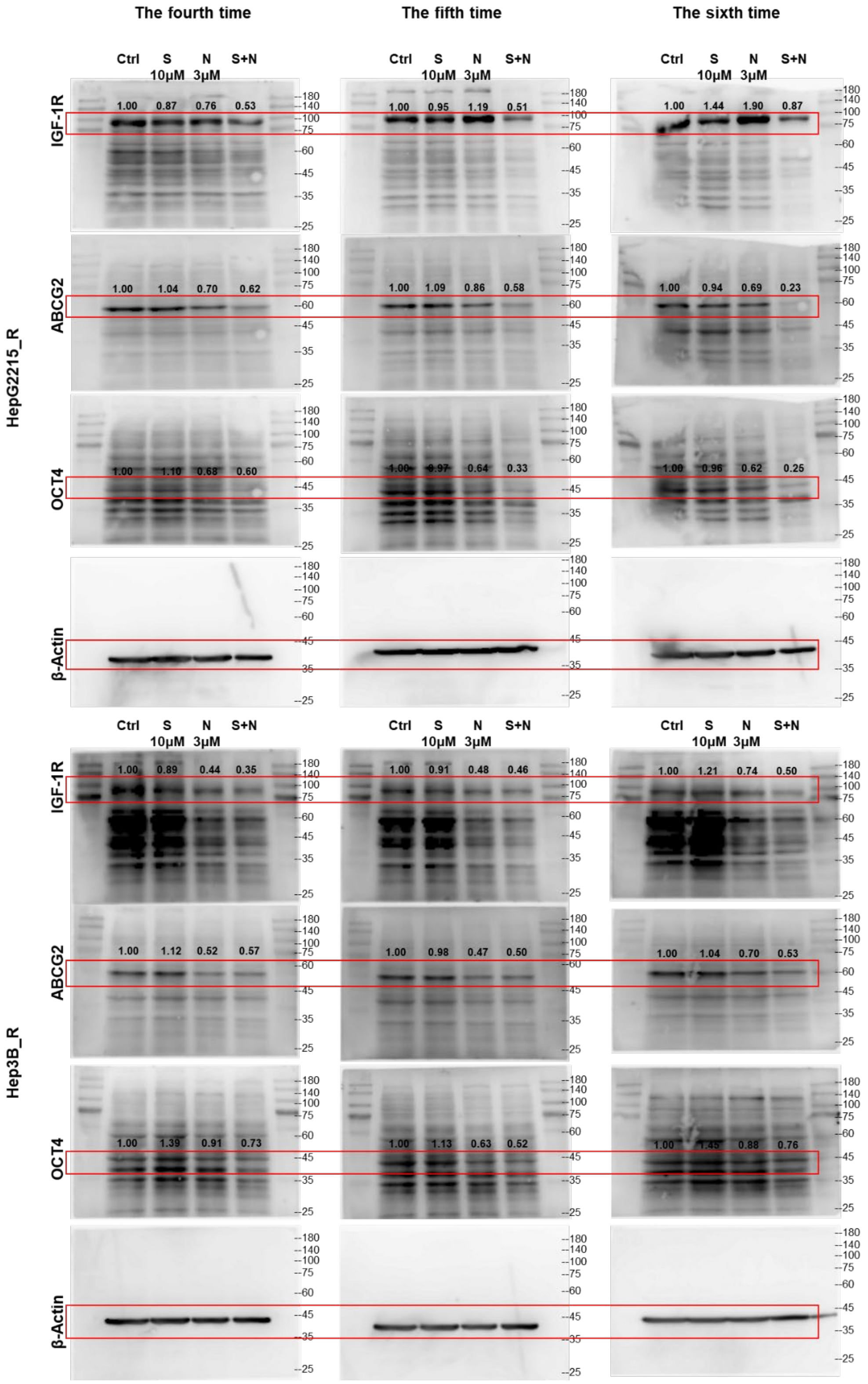

Supplementary Figure S8. The full membrane of western blot results and densitometry readings/intensity ratio for each band of Figure 2B. The protein expression levels of IGF-1R, ABCG2 and OCT4 in sorafenib-resistant HCC cells

(HepG2215_R and Hep3B_R) were shown. The relative quantification was normalized to the corresponding β-Actin.

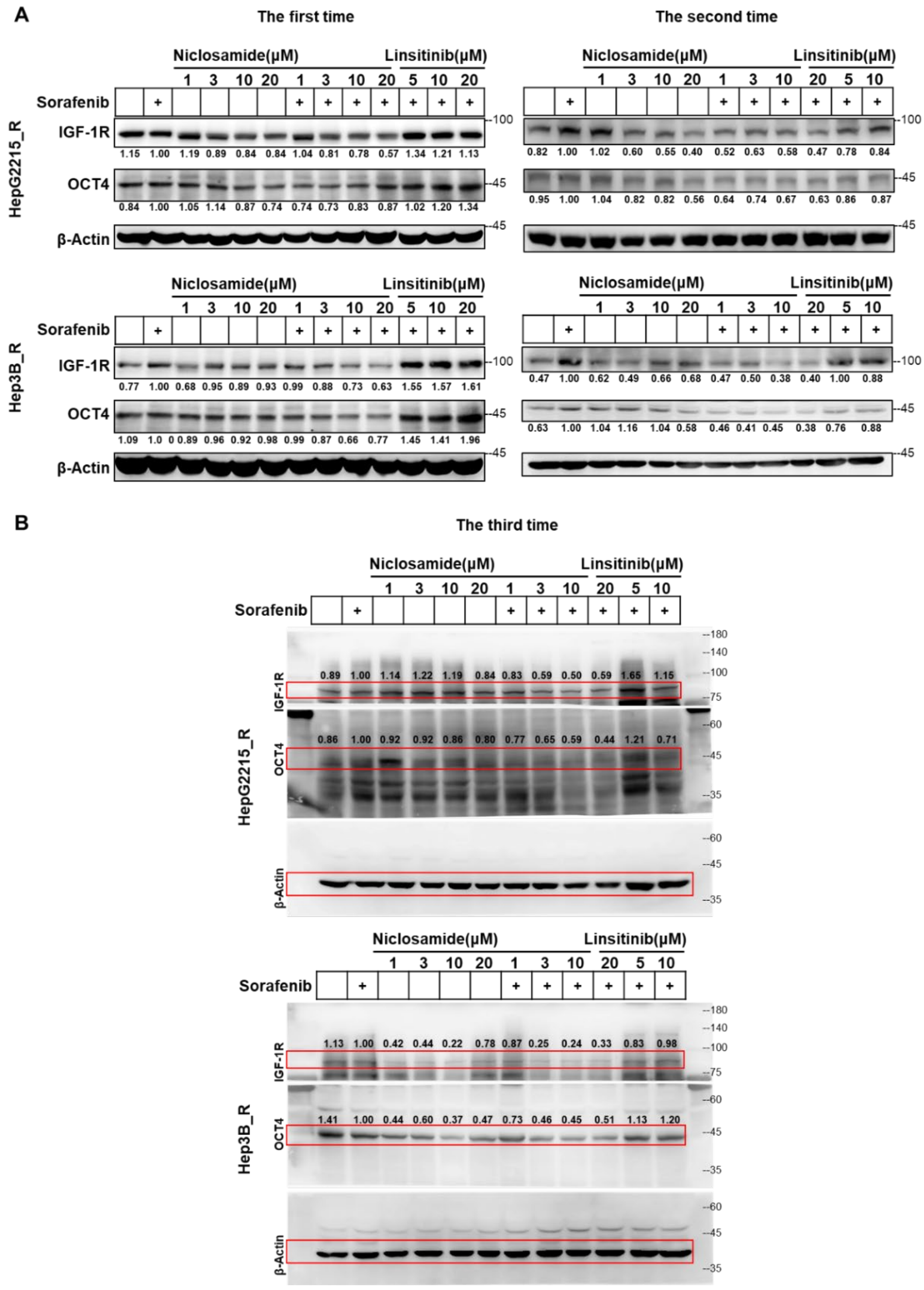

Supplementary Figure S9. The full membrane and triple repeats of western blot analysis and densitometry readings/intensity ratio for each band of main Figure 3A. The protein expression levels of IGF-1R and OCT4 in sorafenib-resistant HCC cells

(HepG2215_R and Hep3B_R) were shown. The relative quantification was normalized to the corresponding β-Actin.

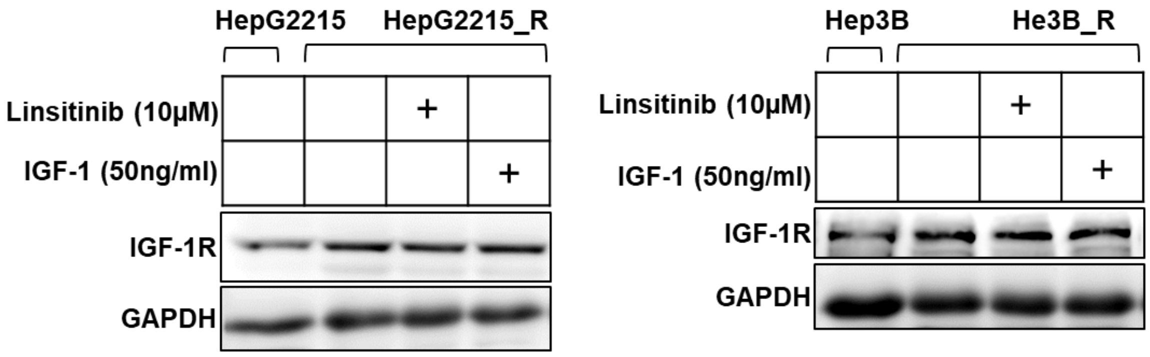

Supplementary Figure S10. Linsinitib alone was unable to decrease the IGF-1R expression levels of sorafenib-resistant HCC cells in 24 h treatment. The protein expression levels of IGF-1R in sorafenib-resistant HCC cells (HepG2215_R and Hep3B_R). The relative quantification was normalized to the corresponding GAPDH.

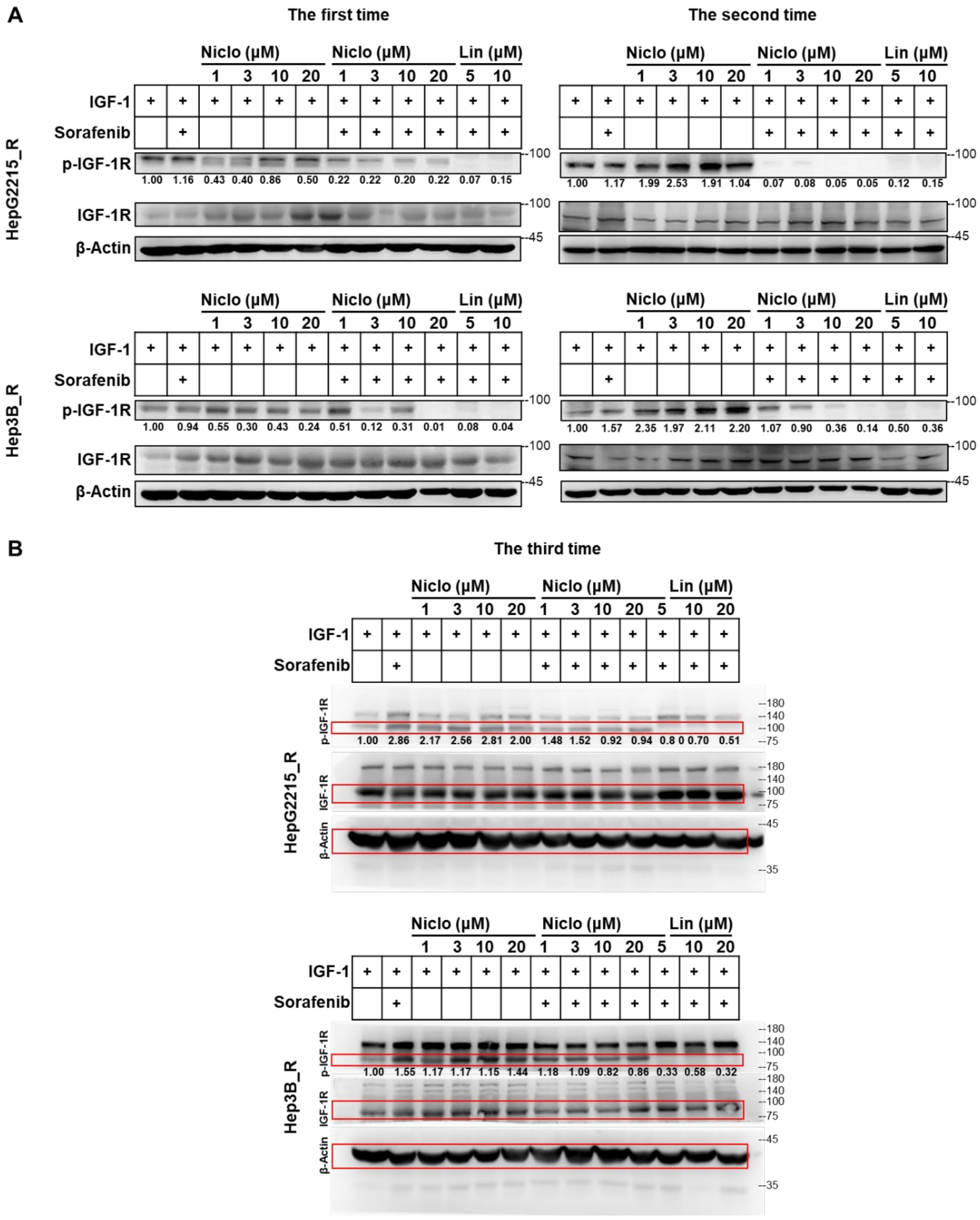

Supplementary Figure S11. The full membrane and triple repeats of western blot analysis and densitometry readings/intensity ratio for each band of main Figure 3D. The protein expression levels of p-IGF-1R in sorafenib-resistant HCC cells

(HepG2215_R and Hep3B_R) were shown. The relative quantification was normalized to the IGF-1R then corresponding β-Actin.

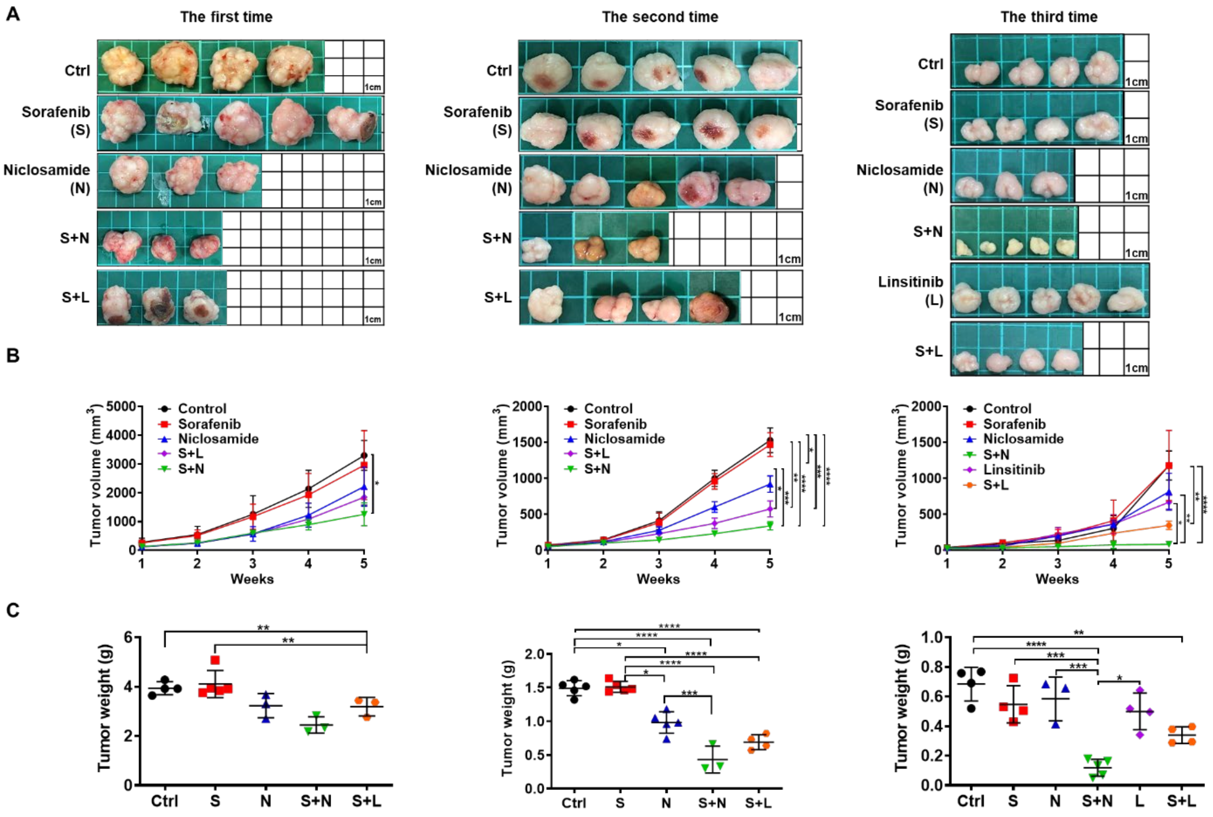

Supplementary Figure S12. The three repeats of animal experiments of Figure 5. **(A)** The size of tumor tissues under different experimental conditions was shown. Control group (Ctrl), sorafenib alone (S, 30 mg/kg), niclosamide alone (N, 40 mg/kg), niclosamide plus sorafenib (S + N), linsitinib (L, 20 mg/kg), and linsitinib plus sorafenib (S + L). **(B)** Tumor volume analysis. **(C)** Tumor weight analysis. **p* < 0.05, ***p* < 0.01, ****p* < 0.001, and *****p* < 0.0001, as obtained from one-way ANOVA.

2. Supplemental Tables

Supplementary Table S1. The list of real-time PCR primers and their product size.

Gene	Accession	Primer	Sequence	Product Size (bp)
IGF-1R	NM_001291858.2	Forward<br>Reverse	CTCCTGTTTCTCTCCGCCG<br>ATAGTCGTTGCGGATGTCGAT	84
IGF-1	NM_000618.5	Forward<br>Reverse	AAAGAAACCTCTCACAGATAAG A<br>AATAATAAGGGCTGGGTTGGGAT	89
IGF-2	NM_001007139.6	Forward<br>Reverse	CACGTCCCTCTCGGACTTG<br>GTGGCATCGTTGAGGAGTG	92
VIMENTIN	NM_003380.5	Forward<br>Reverse	GAGAACTTTGCCGTTGAAGC<br>TCCAGCAGCTTCCTGTAGGT	170
N-CAD	NM_001308176.2	Forward<br>Reverse	GGTGGAGGAGAAGAAGACCAG<br>GGCATCAGGCTCCACAGT	72
E-CAD	NM_001317185.2	Forward<br>Reverse	CCCGGGACAACGTTTATTAC<br>GCTGGCTCAAGTCAAAGTCC	72
ABCG2	NM_ 004827.3	Forward<br>Reverse	GATGTCTAAGCAGGGACGAACA A<br>GGTGAGGCTATCAAACAACTTGA A	82
OCT4	NM_002701	Forward<br>Reverse	CAACTCCGATGGGGCCT<br>CTTCAGGAGCTTGGCAAATTG	148
NANOG	NM_024865	Forward<br>Reverse	CCTGTGATTTGTGGGCCTG<br>GACAGTCTCCGTGTGAGGCAT	114
SOX2	NM_003106	Forward<br>Reverse	GTATCAGGAGTTGTCAAGGCAGA G<br>TCCTAGTCTTAAAGAGGCAGCAA A	77
CD44	NM_000610.4	Forward<br>Reverse	TGCCGCTTTGCAGGTGTATT<br>CCGATGCTCAGAGCTTTCTCC	137
β-2M	NM_004048	Forward<br>Reverse	GTCTCGCTCCGTGGCCTTA<br>TGAATCTTTGGAGTACGCTGGAT A	80

GLUT1	NM_032259.4	Forward	TCACTGTGCTCCTGGTTCTG	70
		Reverse	CCTGTGCTCCTGAGAGATCC	
LDHA	NM_005566.4	Forward	ACGTCAGCAAGAGGGAGAAA	191
		Reverse	CGCTTCCAATAACACGGTTT	
PEPCK	NM_004563.4	Forward	TAGCACCCTCATCTGGGAATA	101
		Reverse	GTCTTTGTGGGAAGGTCTATGG	
HK2	NM_000189.5	Forward	CAAAGTGACAGTGGGTGTGG	87
		Reverse	GCCAGGTCCTTCACTGTCTC	

Supplementary Table S2. List of antibodies.

Protein	Assay	Cat. No.	Company	Origin	Dilution	Incubation Period
IGF-1R	WB	SC-713	Santa Cruz	Rabbit	1:1000	Overnight, 4°C
p-IGF1Rβ	WB	ab39398	abcam	Rabbit	1:1000	Overnight, 4°C
OCT4	WB	sc-5279	Santa Cruz	Mouse	1:500	Overnight, 4°C
ABCG2	WB	NBP2-22124	NOVUS	Mouse	1:1000	Overnight, 4°C
4G10	WB	#96215	Cell Signaling	Mouse	1:1000	Overnight, 4°C
p-PDGFR	WB	#2227	Cell Signaling	Rabbit	1:1000	Overnight, 4°C
PDGRF	WB	#3169	Cell Signaling	Rabbit	1:1000	Overnight, 4°C
p-VEGFR	WB	#4991	Cell Signaling	Rabbit	1:1000	Overnight, 4°C
VEGFR	WB	#2479	Cell Signaling	Rabbit	1:1000	Overnight, 4°C
β-actin	WB	sc-47778	Santa Cruz	Mouse	1:1000	Overnight, 4°C
CK18	IHC	GA618	Dako Omnis	Mouse	1:100	Overnight, 4°C
CK7	IHC	GA618	Dako Omnis	Mouse	1:100	Overnight, 4°C
Ki67	IHC	ab15580	abcam	Rabbit	1:200	Overnight,4°C
Cleaved Caspase-3	IHC	#9661	Cell Signaling	Rabbit	1:100	Overnight,4°C
Control IgG	IHC	#76780	Jackson Labs	Mouse	1:250	Overnight, 4°C

Supplementary Table S3. The detailed data of Figure 3B and 3C.

**HepG2215_R**				**Hep3B_R**			
Niclo(μM)	Sora (μM)	Linsi(μM)	CI index	Niclo(μM)	Sora (μM)	Linsi(μM)	CI index
IC_{50}:15.08	IC_{50}:16.86	IC_{50}:43.95		IC_{50}:17.50	IC_{50}:15.76	IC_{50}:30.80	
1	0.5		0.062	1	0.5		0.113
3	0.5		0.123	3	0.5		0.231
5	0.5		0.183	5	0.5		0.331
10	0.5		0.327	10	0.5		0.603
15	0.5		0.467	15	0.5		0.800
20	0.5		0.525	20	0.5		0.562
30	0.5		0.721	30	0.5		0.744
40	0.5		0.553	40	0.5		0.865
1	2.5		0.148	1	2.5		0.269
3	2.5		0.195	3	2.5		0.403
5	2.5		0.243	5	2.5		0.415
10	2.5		0.354	10	2.5		0.668
15	2.5		0.472	15	2.5		0.852
20	2.5		0.538	20	2.5		0.651
30	2.5		0.636	30	2.5		0.837
40	2.5		0.494	40	2.5		0.901
1	5		0.329	1	5		0.410
3	5		0.321	3	5		0.470
5	5		0.360	5	5		0.558
10	5		0.476	10	5		0.757
15	5		0.580	15	5		0.894
20	5		0.588	20	5		0.696
30	5		0.699	30	5		0.848
40	5		0.497	40	5		0.852
1	10		0.527	1	10		0.609
3	10		0.533	3	10		0.679
5	10		0.575	5	10		0.756
10	10		0.690	10	10		0.682
15	10		0.748	15	10		0.806
20	10		0.810	20	10		0.800
30	10		0.857	30	10		0.990
40	10		0.545	40	10		0.953
1	15		0.622	1	15		0.742
3	15		0.647	3	15		0.796
5	15		0.648	5	15		0.857
10	15		0.670	10	15		0.746
15	15		0.719	15	15		0.792
20	15		0.714	20	15		0.862
30	15		0.684	30	15		0.989
40	15		0.604	40	15		0.928
1		5	0.627	1		5	1.304
2.5		5	0.686	2.5		5	1.288
5		5	0.553	5		5	0.906
10		1	0.865	10		1	1.821
10		2.5	0.517	10		2.5	0.709
10		5	0.474	10		5	0.600
10		10	0.333	10		10	0.517

*Combination Index (CI) which defines Synergism (CI < 1), Additive Effect (CI = 1) and Antagonism (CI > 1)

3. Supplemental Materials and Methods

3.1 Transwell Migration Assays

Transwell assays were performed using 8-μm pore transwell chambers in 24-well plates (Corning Costar, Cambridge, MA, USA). The upper chambers were seeded with 1 × 10^5 hepatocellular carcinoma (HCC) cells in 100 ◎L of serum-free Dulbecco's modified Eagle medium (DMEM). The lower chambers were filled with 800 ◎L of DMEM medium containing 10% fetal bovine serum. Subsequently, the cells were incubated at 37°C in a 5% CO_2 humidified atmosphere for 24 h. After the upper chambers were swabbed to remove cells that did not migrate, the cells that migrated to the lower chambers were fixed with 3.7% paraformaldehyde in phosphate-buffered saline (PBS) and stained using hematoxylin. The migrated cells were counted under a light microscope in five predetermined fields.

3.2 Secondary Tumor Sphere Formation Assay

HCC cells were seeded in an ultra-low attachment multiple 6-well plate (Corning Costar) at 10^3 cells/well and incubated with DMEM-F12 medium (Invitrogen, Carlsbad, CA, USA) containing 2% B27 supplement (Invitrogen). Media were replenished every 2–3 days. Methyl cellulose (1%, Sigma-Aldrich) was added to prevent cell aggregation, and the individual spheres derived from single cells were confirmed. The spheres were harvested after culturing for 10 days and were dissociated into single cells by treatment with trypsin for the secondary sphere formation. The disassociated cells were treated with different experimental conditions

(control, sorafenib 10 ◎◎◎alone, niclosamide 3 ◎◎ alone, and sorafenib combination with niclosamide) for additional 7 days for secondary sphere formation. After 7 days, spheres with diameter > 75 μm were photographed and counted.

3.3 Immunohistochemistry

Tumor slides were incubated at 65°C for 1 h before deparaffinization by xylene followed by washing with decreasing concentrations of ethanol (from 100% to 60%) and then water. Slides were then soaked in boiled 1% unmasking solution for 30 min. After the slides cooled, they were soaked in 3% H_2O_2 in PBS at room temperature for 15 min. Blocking was achieved using normal Goat serum, 2.5% (ImmPRESS Goat Anti-Rabbit IgG polymer Kit–Vector [MP-7451]) for 1 h before incubation with primary antibodies at 4°C overnight. Slides were incubated with secondary antibody (ImmPRESS Goat Anti-Rabbit IgG polymer Kit–Vector [MP-7451]) for 1 h at room temperature. The DAB Substrate Kit–Vector (SK4100) was used. Nuclei were stained with hematoxylin. Finally, slides were dehydrated using increasing concentrations of ethanol and xylene before being mounted using Malinol reagent.
